# Integrated root phenotypes for improved rice performance under low nitrogen availability

**DOI:** 10.1111/pce.14284

**Published:** 2022-02-23

**Authors:** Ishan Ajmera, Amelia Henry, Ando M. Radanielson, Stephanie P. Klein, Aleksandr Ianevski, Malcolm J. Bennett, Leah R. Band, Jonathan P. Lynch

**Affiliations:** ^1^ Division of Plant and Crop Sciences, School of Biosciences University of Nottingham Sutton Bonington UK; ^2^ Department of Plant Science The Pennsylvania State University University Park Pennsylvania USA; ^3^ Strategic Innovation Platform International Rice Research Institute Los Baños Laguna Philippines; ^4^ Centre for Sustainable Agricultural Systems, Institute for Life Sciences and the Environment, Toowoomba Campus University of Southern Queensland Toowoomba Queensland Australia; ^5^ Institute for Molecular Medicine Finland (FIMM) University of Helsinki Finland; ^6^ Centre for Mathematical Medicine and Biology, School of Mathematical Sciences University of Nottingham Nottingham UK

**Keywords:** functional–structural plant modelling, IR64, nitrogen acquisition, nodal roots, *OpenSimRoot*, *ORYZA_V3*, phene synergism, root system architecture

## Abstract

Greater nitrogen efficiency would substantially reduce the economic, energy and environmental costs of rice production. We hypothesized that synergistic balancing of the costs and benefits for soil exploration among root architectural phenes is beneficial under suboptimal nitrogen availability. An enhanced implementation of the functional–structural model *OpenSimRoot* for rice integrated with the *ORYZA_v3* crop model was used to evaluate the utility of combinations of root architectural phenes, namely nodal root angle, the proportion of smaller diameter nodal roots, nodal root number; and L‐type and S‐type lateral branching densities, for plant growth under low nitrogen. Multiple integrated root phenotypes were identified with greater shoot biomass under low nitrogen than the reference cultivar IR64. The superiority of these phenotypes was due to synergism among root phenes rather than the expected additive effects of phene states. Representative optimal phenotypes were predicted to have up to 80% greater grain yield with low N supply in the rainfed dry direct‐seeded agroecosystem over future weather conditions, compared to IR64. These phenotypes merit consideration as root ideotypes for breeding rice cultivars with improved yield under rainfed dry direct‐seeded conditions with limited nitrogen availability. The importance of phene synergism for the performance of integrated phenotypes has implications for crop breeding.

## Summary Statement

Multiscale mechanistic modelling identified several integrated root phenotypes in rice with superior yield under low N availability. Synergism among root phenes was an important component of phenotypic performance.

## 1 INTRODUCTION

Rice is a primary crop for global food security. With the limited availability of arable land and natural resources, rice productivity must increase dramatically to meet a growing demand (Godfray et al., 2010). Poor soil fertility is a major constraint for rice production (S. M. Haefele et al., 2014). Nitrogen (N) accounts for >50% of all applied fertilizers (Mae, 1997) and ∼10%–20% of the total variable cost of rice production (Dobermann & Fairhurst, 2000). Of all fertilizers, N is most susceptible to loss (Schnier, 1995), which may occur via leaching, run‐off, volatilization and denitrification (Choudhury & Kennedy, 2005; Peng et al., 2011). Low recovery of intensively applied N fertilizers represents a major economic, energy and environmental cost of rice production (Ladha et al., 2005). The development of rice varieties capable of sustaining improved yields with low N inputs (i.e., N efficiency) therefore is an important strategy to empower smallholder low‐input rice production systems, mitigate food insecurity and reduce the environmental impact of intensified rice production systems.

Rice is cultivated in diverse agroecologies with an array of different management practices. Rice is predominantly cultivated in lowland ecosystems, which are often aerobic during the dry season (R. J. Buresh et al., 1989). Transplanting is the dominant crop establishment method for rice, but with increasing shortages of labour and water resources, the practice of direct seeding is increasing. Currently, around 23% of global rice production is direct‐seeded (Farooq et al., 2011). With relatively low water and labour input demand, direct‐seeded rice involves sowing either into dry or moist soil with zero or reduced tillage (i.e., dry direct‐seeded rice [DDSR]), puddled soil surface, or standing water (i.e., wet direct‐seeded rice [WDSR]; Kumar & Ladha, 2011). In Asia, DDSR is mainly adopted in rainfed lowlands, uplands, and flood‐prone regions, while WDSR is more common in irrigated regions (Kumar & Ladha, 2011).

Irrespective of the crop establishment method, water management in rice fields varies from irrigated, either with continuous ponding after seedling stage or frequent alternate wetting and drying, to rainfed. However, the global productivity of rainfed rice (<1.0–2.5 t/ha) is often much lower than that of irrigated rice (4.5–>10 t/ha), primarily due to constrained supply of soil resources (GRiSP, 2013). In the tropics, rice is grown over two distinct cropping seasons, namely dry and wet. Tropical dry seasons are climatically characterized to have higher solar radiation due to less precipitation and also lower atmospheric temperature compared to the wet seasons. Dry seasons thus offer a potential advantage in terms of rice yield performance, particularly if the water supply is optimal (Dobermann et al., 2000; Yang et al., 2008).

In rice fields, N fertilizer (commonly urea) is typically split‐applied (twice or three times) in the growing season (Cassman et al., 1993). Once applied to the soil, urea rapidly converts to nitrate and ammonium. Water management is the key determinant factor influencing the dynamics soil N in rice production systems, that is, the amount and timing of irrigation or rainfall corresponding to the application of N fertilizer strongly affect the fate and form of N available in the soil (S. Haefele et al., 2016). In flooded hypoxic paddies, ammonium remains the predominant form of soil N available to the rice plants, whereas drying of the soil surface increases N loss via volatilization. In contrast, in aerobic soils, typical to DDSR systems, nitrate is the commonly available form of N due to nitrification (Araus et al., 2020). Being highly water‐soluble, nitrate leaches to deeper soil strata in aerobic soils, resulting in the loss of N from the root zone (Choudhury & Kennedy, 2005). Such leaching along with surface soil drying over the growing period, particularly in tropical dry seasons, enhance the movement of mobile resources, including N, to deeper soil domains. Unlike flooded paddies in which N losses via denitrification and volatilization are prevalent, and the hardpan restricts the downward flow of water along with N, leaching poses a major factor contributing to N losses in aerobic soils (Araus et al., 2020; R. Buresh et al., 2008).

One avenue to breed N‐efficient crops including rice is the selection for cultivars with root phenotypes that permit superior soil exploration and N capture (Dathe et al., 2016; Henry, 2013; Lynch & Brown, 2012; Lynch, 1995, 2013, 2015, 2018, 2019, 2021). The rice root system consists of a primary root, several nodal roots with large and small diameters and two main classes of lateral roots: long and thick L‐type and short and small S‐type (Kawata et al., 1963; Kono et al., 1972; Yamauchi et al., 1987). L‐type and S‐type lateral roots appear on all axial roots. L‐type laterals are moderately geotropic with indeterminate growth and bear S‐type laterals. S‐type laterals are ageotropic, growth‐determined and do not bear any secondary roots (Moldenhauer & Gibbons, 2003; Périn et al., 2007; Rebouillat et al., 2009). S‐type laterals are the most numerous, accounting for ∼70%–75% of the total root system (Nestler et al., 2016). With appropriate soil aeration, all root types, except S‐type laterals, bear root hairs at a short distance from the root tip. In the vegetative growth phase, nodal root branching and tillering is well synchronized with leaf emergence (Araki et al., 2000; Nemoto et al., 1995).

The root phenotype is an emergent property of individual phenes (phene is to phenotype as gene is to genotype)—broadly classed as architectural, anatomical and physiological (York et al., 2013). Root system architecture, the spatiotemporal configuration of a root system, is a combination of different architectural phenes such as the number of nodal roots, nodal root growth angle and lateral root branching density. These phenes influence soil resource acquisition by colocating root foraging with resource availability in space and time (Lynch, 2019). Anatomical phenes, such as root cortical aerenchyma, cortical cell size and file number, xylem size and number, and root hair length and density regulate the metabolic cost of soil exploration and resource transport (Lynch, 2015). Furthermore, physiological phenes such as the expression of resource uptake transporters influence soil resource capture (York et al., 2016).

In general, rice has a shallow root system compared to other crops. Deep roots are advantageous for soil N capture across most agroecosystems (Kell, 2011; Lynch, 2013; Thorup‐Kristensen et al., 2020). Deep rooting is an aggregate of multiple distinct phenes, which are under distinct genetic control (Lynch & Wojciechowski, 2015). For example, the DRO1 gene regulates root growth angle, conferring deep rooting in an upland rice variety and in turn influencing N uptake when introgressed into IR64 (Arai‐Sanoh et al., 2014; Uga et al., 2013). Selection for root phenes, such as root angle, or even specific combinations of particular phenes, is more genetically tractable, precise, and less complex than selection for composite root traits such as deep rooting. Thus, strategically combining multiple root phenes by adopting ideotype or trait‐based breeding has great potential for enhancing the efficiency of soil N capture and yield (Lynch, 2019). This approach not only considers specific phenes but also the relationship among the phenes since the integrated phenotype determines plant fitness in terms of crop performance in different agroclimatic and edaphic conditions.

Root phenes interact with each other to determine plant fitness. Interactions among root phenes are either synergistic, additive or antagonistic and their utility for different environmental, management and other plant factors are manifold and complex. This results in multiple root phenotypes being optimal for a given resource regime and across contrasting environments (Rangarajan et al., 2018). Understanding the fitness landscape of integrated root phenotypes (i.e., the relationship between phenes in different states and the performance of the integrated root phenotypes) for the target environment (e.g., low soil N availability in the DDSR agrosystem) is essential for the informed deployment of root phenotypes for crop improvement. The interplay of different phene states and their interactions with the environment leads to a large number of scenarios of interest. Evaluating such a large number of scenarios exceeds the capacity of empirical research. For example, if each of five phenes of interest exists in one of four states (e.g., very steep, steep, intermediate and shallow nodal root growth angle), there exist 4^5^ = 1024 phenotypic combinations, each of which should be evaluated in several environmental scenarios. In reality, root phenes exist in more than four states, and multiple soil environments may be targeted. In this context, functional–structural plant models are a valuable tool (Band et al., 2012; Godin & Sinoquet, 2005; Louarn & Song, 2020; Vos et al., 2010). One such functional‐structural model is *OpenSimRoot*, which simulates the growth and soil resource acquisition of a root system in three dimensions over time (Lynch et al., 1997; Postma et al., 2017). *OpenSimRoot* is the leading tool to evaluate the functional utility of specific root phenes and their interactions with other phenes across different climates, nutrient regimes, and soil types. It has enabled the identification of optimal root ideotypes for nitrogen, phosphorus and potassium acquisition in barley, common bean, maize, lupin, rice and squash (Chen et al., 2011; Dathe et al., 2016; Gonzalez et al., 2021; Postma & Lynch, 2011a, 2011b, 2012; Postma et al., 2014; Rangarajan et al., 2018; Schneider et al., 2017; Walk et al., 2006).

This study presents an enhanced implementation of *OpenSimRoot* for rice to discover optimal root architectural phenotypes for improved performance in rainfed DDSR conditions with low N supply in tropical dry seasons. This production environment corresponds to the low‐input rice farms in South and South‐East Asia where the supply of soil resources is scarce or expensive to favour typical lowland rice production. We hypothesized that synergistic balancing of metabolic costs and soil exploration among architectural root phenes such as nodal root angle, diameter, and number; L‐type and S‐type lateral branching density is beneficial for plant growth under low N. These five root phenes were evaluated in silico, independently and in combinations, for their roles in improving shoot biomass accumulation and yield under low nitrogen supply in rainfed dry direct‐seeded conditions. Sustaining our hypothesis, multiple integrated root phenotypes with substantial synergism among root phenes were predicted to improve low nitrogen tolerance for rainfed DDSR.

## 2 MATERIALS AND METHODS

### 2.1 Model description

#### 2.1.1 Background

*OpenSimRoot*, a functional–structural modelling platform (Postma et al., 2017), was used to simulate root growth, root–soil interactions, soil resource capture, and growth of a rice plant under varying soil nitrogen availability. *OpenSimRoot* is an open‐source, extended version of *SimRoot* (Lynch et al., 1997) as described in (Postma et al., 2017). *OpenSimRoot* represents a plant as hierarchically interacting components (mini/submodels) which predict the function of root phenotypes in a whole‐plant context. It simulates individual root classes consisting of discrete small (∼1 cm) connected root segments, leading to a whole root system architecture. The model dynamically simulates root growth and soil resource acquisition in three‐dimensional virtual soil. A finite element model is used to simulate the soil domain such that each node/element holds the values for nutrient and water content, and several soil properties. This finite element model captures unsaturated water flow and solute transport in the soil domain, including root uptake, by using the Richards equation (Richards, 1931) for water and the convection‐dispersion equation for solutes. In the soil domain, the initial nitrate in the topsoil leaches to the deeper domain over time depending on the precipitation events. The soil domain also incorporates the nitrate mineralization model (Yang & Janssen, 2000), which is independently simulated for each finite element node.

The model explicitly accounts for total metabolic costs of root growth, maintenance, and nutrient (nitrogen, phosphorus and potassium) capture. Nitrogen uptake is simulated using Michaelis Menten kinetics (Barber, 1995) wherein the nitrogen concentration at the root surface is the average nitrogen concentration of finite element nodes surrounding the root. The root water uptake model based on Alm's equation has been adapted for growing root systems (Alm et al., 1992). The model accounts for axial and radial hydraulic conductivity for individual root segments across the root system. Root water and nitrate uptake is lumped into respective sink terms. Discrepancies between source and sink strength for the acquired carbon and nutrients are settled using a set of empirically derived growth responses. Total nutrient acquisition by the root system is calculated by integrating nutrient uptake overall across root segments. Shoot growth is nongeometrically represented by a canopy model factoring the shading effect and simulating leaf area index, light capture, and gas exchange. Each simulation depicts a growing plant for a given planting density defined by inter‐ and intrarow spacing. The boundary conditions at the mid‐distance between the adjacent plants were defined such that zero‐flux occurred across the boundaries and the roots were mirrored back in, to simulate a field‐like root density distribution. To simulate rice plant growth, we have updated the previous tillering module in *OpenSimRoot* (Gonzalez et al., 2021; Schneider et al., 2017) by incorporating different orders of tillers, namely primary, secondary and tertiary, thereby accounting for roots that originate from each tiller.

#### 2.1.2 Assumptions and parameterization

Being a heuristic model, the primary purpose of *OpenSimRoot* is to test the adequacy of a hypothesis to explain the observed results. *OpenSimRoot* involves an extensive set of empirical parameters, which can be broadly classed into six categories: root morphology, plant physiology, root anatomy, growth kinematics, weather and soil environment and planting scheme. Importantly, the model is never calibrated to force agreement with empirical results, as this would violate the premise of heuristic modelling. In the current study, most parameters are adapted from previously published rice literature, particularly corresponding to cv. IR64 (Supporting Information 1). Unlike previous rice root system models (De Bauw et al., 2020; Gonzalez et al., 2021) wherein the root growth is limited only by the availability of a soil resource, the current model simulates root growth which is limited by both photosynthates and the availability of soil resources over plausible soil and climatic conditions. The model assumes that ammonium is readily converted to nitrate, and soil temperature and root exudation is currently ignored. Moreover, the exponential decline in the soil carbon pool via aging with decreasing breakdown rate was assumed in the model, whereby the C:N ratios of the organic substrate and microbial biomass lead to the estimation of the net mineralization of N. Soil N availability in the model corresponds to fertilization and mineralization of organic matter.

Parameters associated with each root class were assumed to be identical when root class‐specific measurements were lacking. All the evaluated root phenotypes are assumed to be ‘nonplastic’ in response to local soil N availability. Under this assumption, only apparent root plasticity (i.e., those lacking adaptive value—e.g., reduction in shoot and root growth in response to low N) is captured while the adaptive plasticity is set to null (i.e., those associated with the plant fitness—e.g., nodal root number or lateral densities—do not alter in response to low N; Schneider & Lynch, 2020). It is assumed that all nodal roots emerge at a similar angle with a stochastic gravitropic set point angle and some degree of stochastic soil impedance on root growth to have a more realistic distribution of roots in the soil profile. Moreover, it is assumed that there is no variation in L‐type and S‐type lateral root densities across large and small diameter nodal roots. Root growth rates decreased over time, and different root classes and developmental stages were considered. No replicate simulations were performed due to the computational constraints in simulating a dense root system for rice. Furthermore, variation in the phenotype response is largely constrained as most architectural root phenes are precisely set for evaluation, which in turn trivializes the need for performing replicate simulations. The full parameter set, with references, is provided in Supporting Information 1.

#### 2.1.3 Simulated scenarios and environments

A single rice plant was simulated representing a monoculture field with a between‐row spacing of 25 cm, within‐row spacing of 20 cm and soil depth profile of 80 cm, corresponding to the planting density of 20 plants/m^2^. At the start of the simulation, a seed was sown at the depth of 2.5 cm in the middle of a virtual soil column of dimension 25 × 20 × 80 cm. Roots from neighbouring plants were simulated by mirroring the roots at the boundary back into the column (Postma & Lynch, 2011a). The N fertilizer applied corresponds to the first of three split N doses, typically 60–60–60 kg N/ha, prescribed over the vegetative growth stage. Although nitrate fertilizers are typically given in split dose in rice agroecologies, we focused our study on the first 30 days after germination (DAG) of root system growth, which would normally be before the application of the second dose.

To accurately mimic the field environment, soil and weather parameters were based on conditions measured at International Rice Research Institute (IRRI) headquarters, Los Baños, Philippines. Nonpuddled soil environments to capture DDSR were simulated in *OpenSimRoot* by adapting the set of soil parameters from the *ORYZA_v3* model (Li et al., 2017). Climate and precipitation data for the dry season beginning on January 2016 from the IRRI weather station were used in all simulations across this study (see Supporting Information 1). Water deficit is not implemented in this model, and as a result any variation in the soil water level only alters the soil nitrogen dynamics but has no direct influence of plant growth.

The *OpenSimRoot‐Rice* model developed for the IR64 cultivar was simulated over these production systems with four different soil nitrogen availabilities (i.e., 5.8, 11.6, 29 and 58 kg/ha in the soil solution) and the effect of individual root phene states on plant growth in response to different soil N availabilities under rainfed dry direct‐seeded conditions was evaluated. To identity optimal phenotypes with improved tolerance to low N supply, 1024 root phenotypes generated in a full factorial design fashion were simulated in rainfed dry direct‐seeded conditions over the dry growing season with low and optimal soil N supply (i.e., 5.8 and 58 kg/ha). Details of all these virtual experiments are described in Table 1.

**Table 1:** Root phene combinations and experimental design used in the simulations of rice root architecture

Root traits	Varied levels	Units	No. of levels
Nodal root angle	***30***, 45, 60, 80	Degree	4
Nodal root diameter	20, ***32***, 68, 100	% Of small diameter nodal roots	4
Nodal root number	0.65, ***1***, 1.35, 1.69	Fold	4
Lateral root branching density	2, 5, ***10***, 20	per 10 cm	4
Fine lateral root branching density	10, 50, ***100***,150	per 10 cm	4
Treatment			
Soil nitrogen supply	5.8 & 58	kg N/ha (in liquid phase)	2

*Note*: Bold indicates reference phenes from IR64.

#### 2.1.4 Estimating phene interactions

To quantify the type of phene interaction (synergism, antagonism or additivity) for each phenotype, the observed response of integrated phenotypes was compared to the expected response under the assumption of nonsynergism using the conventional additive null model (Côté et al., 2016; Ianevski et al., 2020; Ma et al., 2001). Here, the response is the simulated shoot biomass under low N. The response of individual phene states was obtained by subtracting the reference phenotype response from that of each phene combination response differing in just a single phene state. The expected additive response for different phene combinations was calculated by summing the corresponding individual phene state responses. The obtained expected responses of integrated phenotypes were than summed with the reference phenotype response, to be comparable with actual responses (Figures 6 and 8). The difference between the actual and expected response indicates the type of phene interaction, wherein the positive and negative values respectively depict synergistic and antagonistic phene combinations, while those with smaller difference (i.e., <±0.05 g) were classed as additive or noninteractive. Technically, the synergy estimation method implemented here is an adapted version of the formalism used for quantifying synergies among multidrug combinations such that drug and drug concentrations represent phene and phene states (Ianevski et al., 2020). The corresponding R script, input and output files can be found in Supporting Information 2.

#### 2.1.5 K‐prototype clustering

The simulated phenotypes with shoot biomass reduction of ≤45% in response to low N availability derived from the full factorial experiments were screened for significant phenotypic differences. Root phene states occurring at high frequency are likely to be consistently related to the corresponding phenotypic performance under low N. The defined phene states are categorical in nature while the corresponding shoot biomass gain is continuous, leading to a mixed‐type data set. The k‐prototype method for clustering mixed‐type data were implemented using the clustMixType package in R (Szepannek, 2019) to identify clusters of phene state combinations most commonly associated with improved shoot biomass accumulation under low N availability.

#### 2.1.6 Coupling *OpenSimRoot/Rice* with *ORYZA_v3*

*ORYZA_v3*—an advanced version of *ORYZA2000* (Bouman et al., 2001; Li et al., 2017), is an ecophysiological crop model simulating rice development, growth, and yield under various environmental conditions, including water‐ and nitrogen‐limited conditions. It has been extensively calibrated and validated across different rice ecosystems and varieties (Bouman & van Laar, 2006; Li et al., 2013; Radanielson et al., 2018). To simulate the effects of root phenotypes on crop yield, the *ORYZA* model was set to use the input simulations over the vegetative stage (i.e., 30 DAG) of biomass and leaf growth from *OpenSimRoot/Rice*. Like *OpenSimRoot*, *ORYZA* models were set to simulate the environmental conditions for the 2016 dry growing season from the IRRI headquarters research station, Los Baños, Philippines. Two nitrogen application regimes for rainfed DDSR over dry growing season were considered, namely low and high correspond to the total nitrogen fertilization of 17.4 and 174 kg/ha split applied as three equal doses over the growing season (i.e., 5.8–5.8–5.8 and 58–58–58 kg/ha). The Crop was sown with a planting density of 20 plants/m^2^. Carbon partitioning and leaf area index simulated by *OpenSimRoot/Rice* for the reference cv IR64 and predicted phenotypes under optimal and low N supply over 30 DAG were used as inputs to the *ORYZA_V3* model to generate forecasts of final grain yield. The coupled model was used to predict the performance of each phenotype simulated over the past 33 years of historical weather and future 80 years of predicted weather. The future weather data were obtained from climate projections provided by CIMP6 (O'Neill et al., 2016) considering climate change scenarios under a more sustainable pathway of global trends and a conservative global temperature rise below 2°C. A step‐by‐step description on this coupling and model calibration is presented as Supporting Information 3.

## 3 RESULTS

**Figure 1:**
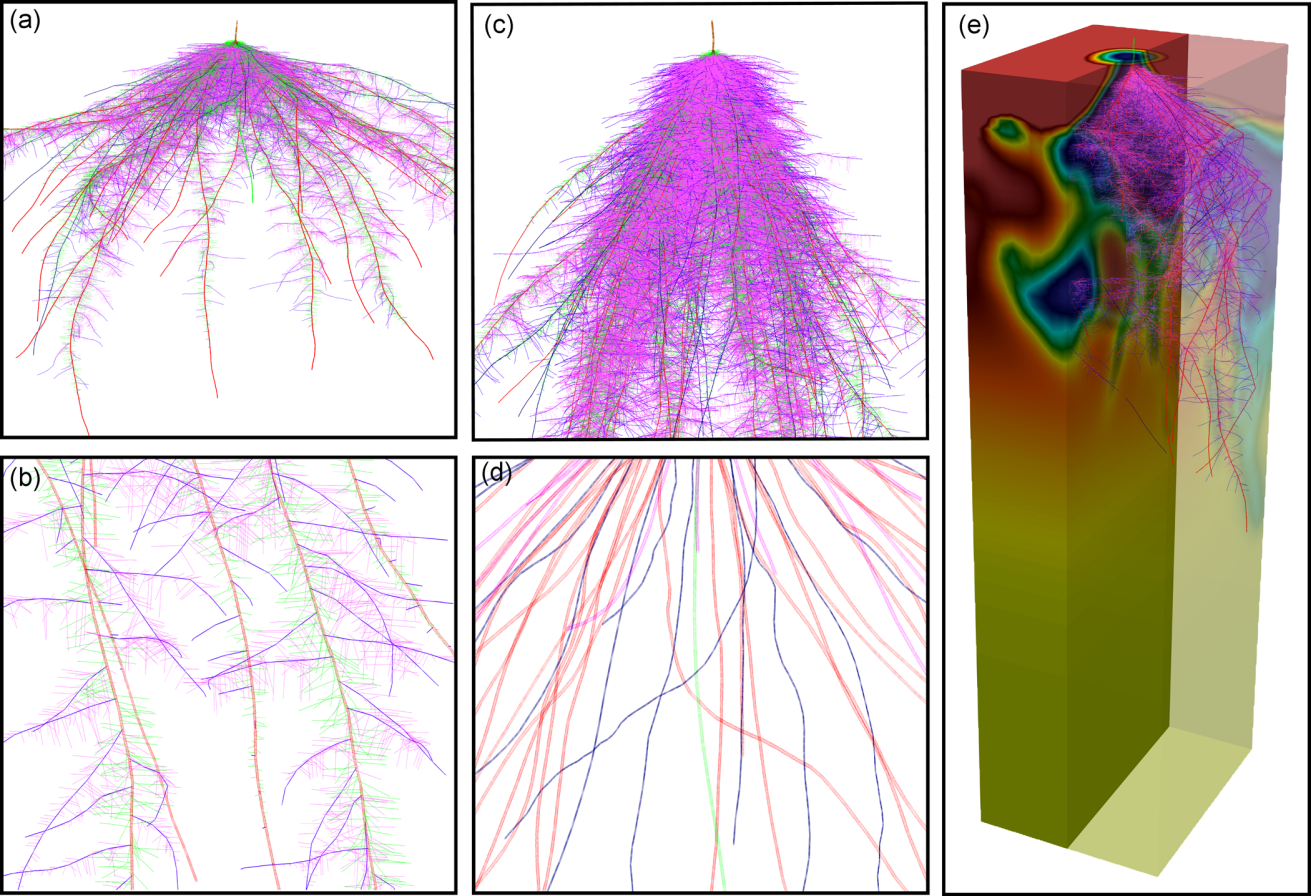
Visualization of different morphological root phenes simulated in *OpenSimRoot/Rice*. Panel (a, c) depicts rice root system with shallow (30°, panel a) and steep (60° panel c) nodal root angle. Panel b depicts different root classes—nodal (red), L‐type laterals (blue), S‐type laterals on nodal (green) and on L‐type laterals (pink). Panel d depicts different axial roots in a root system without any laterals—primary (green), large (red) and small (blue) diameter nodal roots and nodal roots from tillers (pink). Panel e highlights soil nitrogen dynamics with IR64 root system at 30 DAG, wherein red to dark blue colour depicts highest to lowest nitrogen availability in the soil, respectively. DAG, days after germination [Color figure can be viewed at wileyonlinelibrary.com]

**Figure 2:**
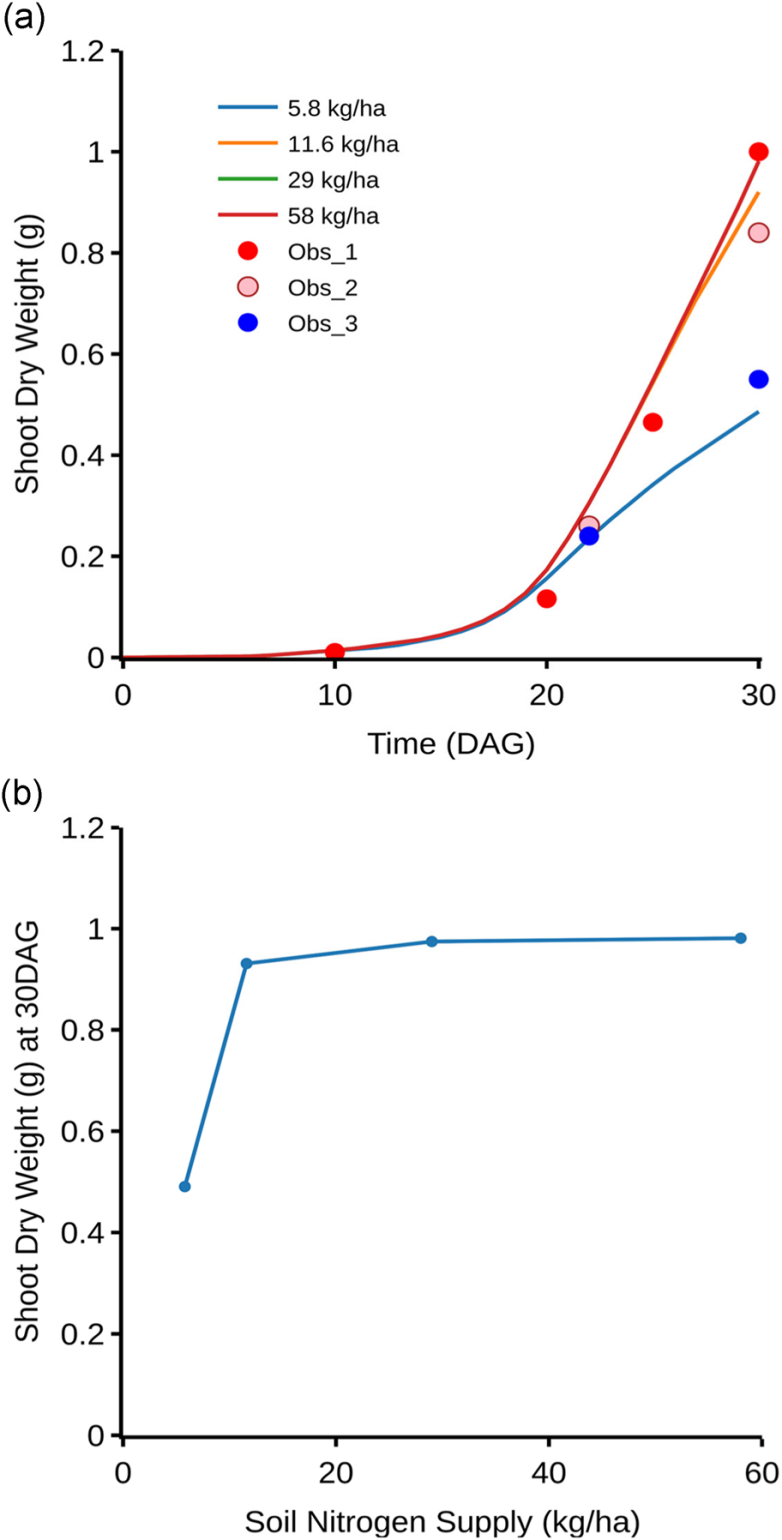
Shoot biomass of the reference phenotype (IR64) over 30 days with different initial soil nitrogen supply. (a) Simulated versus observed shoot growth under optimal and low N conditions. Obs1, 2 & 3 are the measured shoot dry weights for rice cv. IR64 over optimal and low N supply (Luquet et al., 2006); (b) simulated shoot growth at 30 DAG with varying N supply under rainfed dry directed seed condition over dry growing season. DAG, days after germination [Color figure can be viewed at wileyonlinelibrary.com]

Development of *OpenSimRoot/Rice* for this study permitted the evaluation and visualization of how variation in five root architectural phenes and their resulting integrated root phenotypes affected shoot growth in response to soil nitrogen dynamics (Figure 1, see Supporting Information 4 for simulation video). *OpenSimRoot*/*Rice* accurately simulated the observed shoot growth of rice cv. IR64 over 30 DAG under both optimal and low N supply (Figure 2a). In rainfed dry direct‐seeded conditions over the dry season, shoot growth of rice *cv*. IR64 over 30 DAG was predicted to be reduced by 50.6% at 5.8 kg N/ha, by 5.6% at 11.6 kg N/ha and optimal at >29 kg N/ha (Figure 2b).

**Figure 3:**
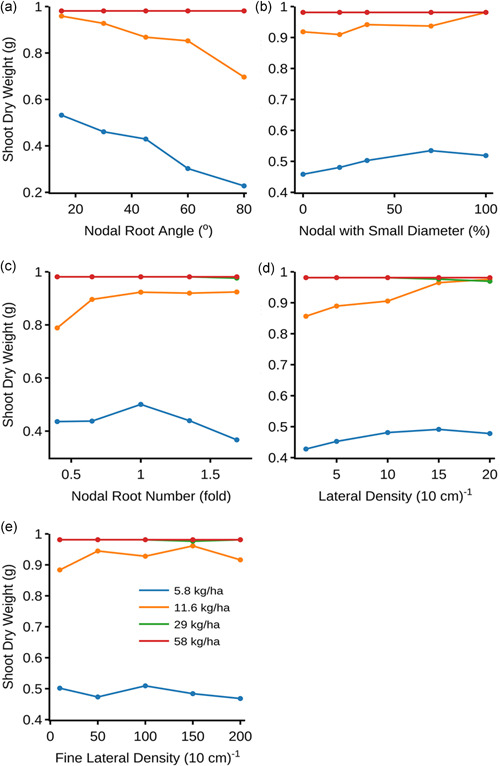
Shoot biomass over (a) varied nodal root angle (b) nodal root diameter (c) nodal root number (d) L‐type lateral root branching density (e) S‐type lateral root branching density under four levels of soil nitrogen supply [Color figure can be viewed at wileyonlinelibrary.com]

Individual root phenes influenced shoot biomass production at 30 DAG under suboptimal N availability (Figure 3). The reference phenotype was parameterized to represent a rice root system of the widely‐grown variety IR64, with shallow nodal root angle (30°), relatively low proportion (i.e., 32%) of smaller diameter nodal roots from the main stem, 44 nodal roots from the main stem (termed as reference nodal root number), and L‐type and S‐type lateral densities of 1 and 10 roots per cm, respectively. In low N, shoot biomass was increased by adjusting the root phenotypes with either shallower nodal root angle (Figure 3a), or a greater proportion of smaller diameter nodal roots (Figure 3b). Setting the number of nodal roots similar to the reference phenotype showed greater shoot biomass than phenotypes with fewer or more nodal roots under low N availability (Figure 3c). Greater lateral root density (i.e., L‐types) showed greater shoot biomass under both low and intermediate N supply (Figure 3d), while fine lateral root densities (i.e., S‐types) had little effect on shoot biomass under low N (Figure 3e).

**Figure 4:**
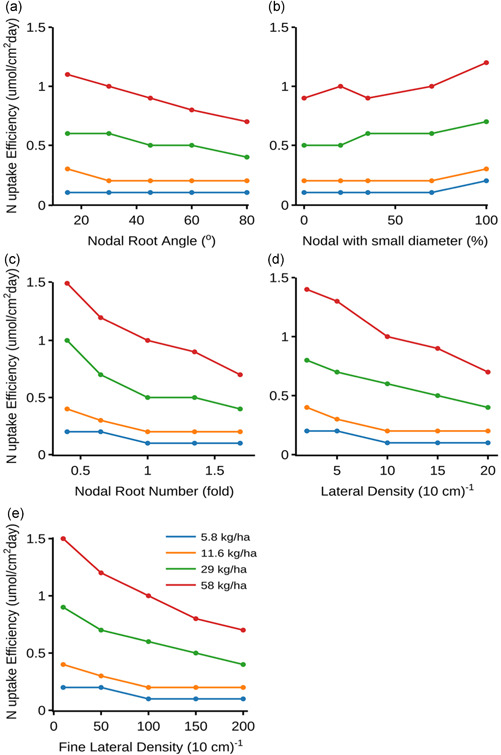
Nitrogen uptake efficiency (μmol/[cm^2^CCday]) of the phenotype at 30 DAG with varying individual root phenes over different initial soil N supply (same as Figure 3). The N uptake efficiency is calculated by dividing the cumulative N uptake (μmol) over 30 days with the root surface area duration (${\mathrm{RSA}}_{\mathrm{dur}}$, cm^2^·day)—an integral of the root surface area over time. The latter was as the sum of the areas of a series of rectangles. The area of each rectangle is the product of the interval between two consecutive time points and the average of the root surface area at each of the two time points. ${\mathrm{RSA}}_{\mathrm{dur}}\approx \sum_{t=1}^{t=30}\frac{\left({\mathrm{SA}}_{t}-{\mathrm{SA}}_{t-1}\right)}{2}\times A_{t}-A_{t-1}$ Where, *t* are the time points, $A_{t}$ is age of plant at time *t*, and ${\mathrm{SA}}_{t}$ is root surface area at time *t*. This is equivalent to the trapezoid rule in numerical integration. Root competition and poor coincidences of roots and soil resource in space and time may decrease resource uptake per unit area. DAG, days after germination [Color figure can be viewed at wileyonlinelibrary.com]

**Figure 5:**
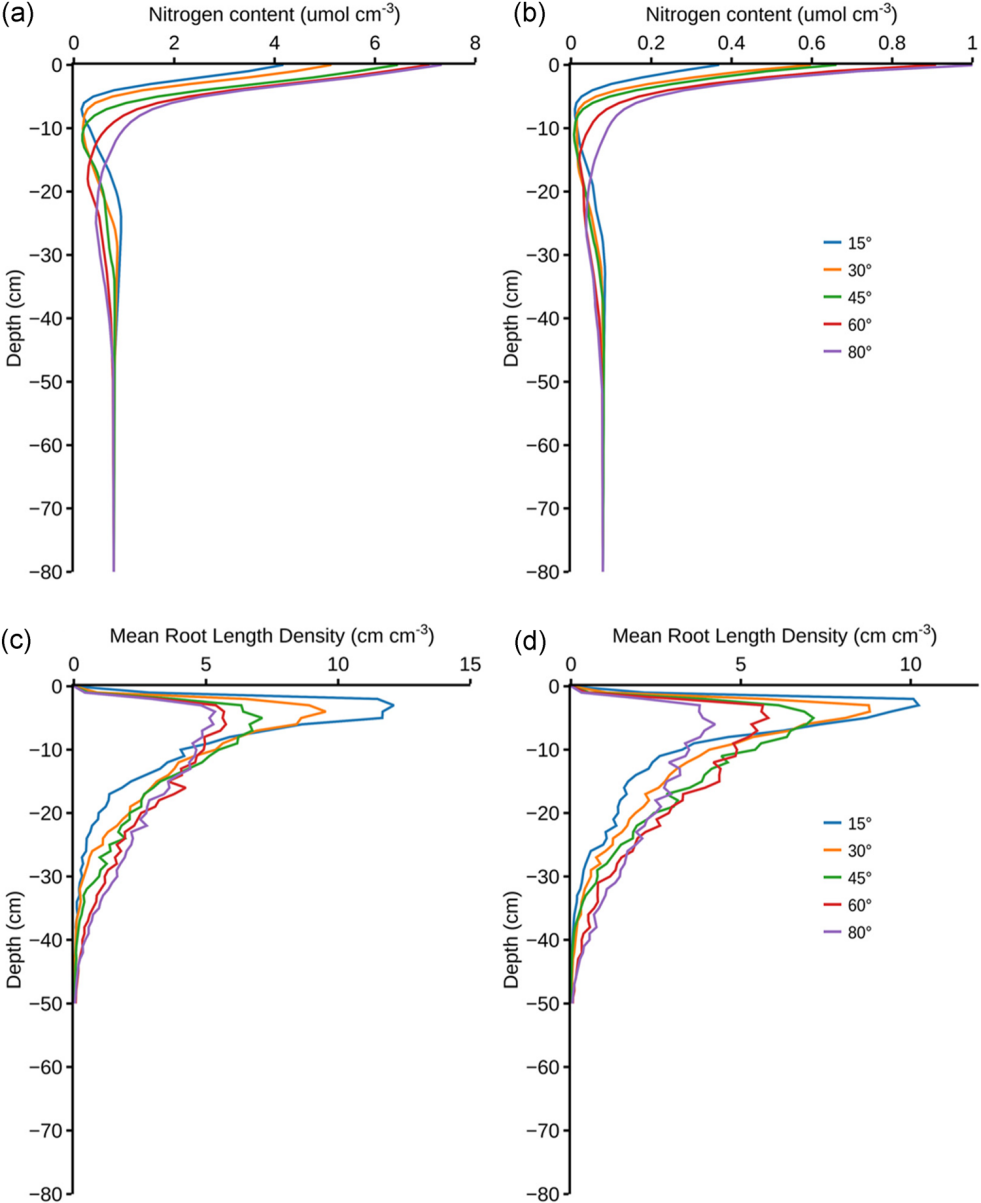
Nitrogen content and root length density over soil depth at 30 DAG for root system with varying nodal root angle over (a, c) optimal (58 kg/ha) and (b, d) low (5.8 kg/ha) initial soil N supply. DAG, days after germination [Color figure can be viewed at wileyonlinelibrary.com]

Intraplant root competition influences resource capture of the entire root system (Postma & Lynch, 2012; Rubio et al., 2001; Schenk, 2006; York et al., 2013). To evaluate how effective the individual root segments were at acquiring soil N in different root phenotypes, the N uptake per unit root surface duration, representing N uptake efficiency, was computed (Figure 4). Increasing the number of nodal roots and lateral branching densities (both L‐type and S‐type) decreased the soil N uptake efficiency (Figure 4c–e). On other hand, an increase in the proportion of small diameter nodal roots was associated with an increase in N uptake efficiency, particularly under optimal N supply (Figure 4b). Shallower root systems acquired N more efficiently irrespective of the initial N supply (Figure 4a). Correspondingly, N availability in shallower soil layers decreased with increasing shallowness of the root systems over 30 DAG with both low and optimal N supply (Figure 5a,b).

The functional utility of an integrated phenotype is influenced by the interactions among the corresponding phene states (Rangarajan et al., 2018). *OpenSimRoot/Rice* enabled screening of multiple root phenotypes generated in silico by integrating these five architectural phenes varied at four different levels in a factorial design (Table 1). The shoot biomass of the reference phenotype was reduced by 50.6% under low N supply, while the reduction in shoot biomass of the simulated integrated phenotypes in response to low N supply ranged from 35% to 92%. Multiple integrated phenotypes (i.e., 147) resulted in improved plant performance under low N with shoot biomass reduction of less than the reference value of 50.6%. Likewise, a large array of integrated phenotypes (i.e., 876) had inferior performance under low N, with more than 50.6% reduction in shoot biomass.

**Figure 6:**
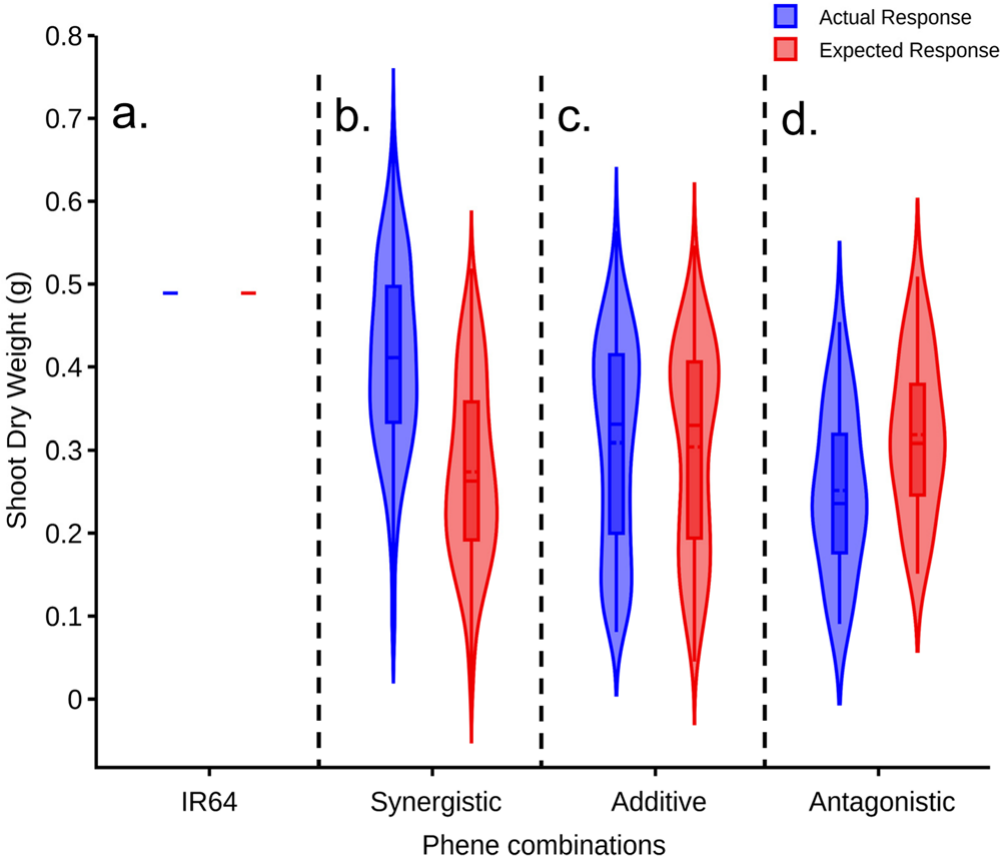
Reference phenotype IR64 (a), synergistic (b), additive (c), and antagonistic (d) effects of 1024 simulated phene combinations on shoot dry weight under low N conditions. Actual responses (i.e., shoot biomass, blue) are greater than, equal to and less than the expected (red) for synergistic, additive and antagonistic phenotypes, respectively [Color figure can be viewed at wileyonlinelibrary.com]

Phene synergism occurs when the benefits of a certain phene combination are greater than the expected additive effects alone, while the opposite (i.e., less fitness than the expected additive effects) is caused by phene antagonisms. The expected additive responses for 1024 integrated phenotypes under low N were quantified, based on the reference phenotype response (Supporting Information 2). Of these, 451 integrated phenotypes showed synergism with 0.4–0.05 g greater shoot biomass than would be expected by the additive effects of their phene states (Figure 6b). This includes 147 phenotypes with greater shoot biomass than IR64 in response to low N (Figure 6a,b). For 531 integrated phenotypes, the difference between the shoot biomass and their additive expectation was trivial (i.e., within ±0.05g) and were thus classed as additive or noninteractive combinations (Figure 6c). The remaining 42 integrated phenotypes showed antagonistic interactions, with 0.05–1.2 g less shoot biomass than their expected additive response (Figure 6d).

**Figure 7:**
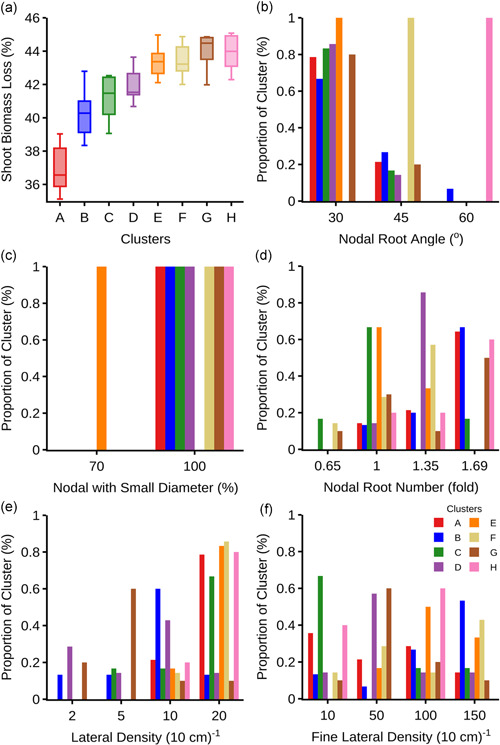
k‐Proto clustering of 70 root phenotypes with threshold reduction in shoot dry weight (i.e., ≤45% [a]) in response to low soil N availability. Clustering led to eight distinct clusters, sequentially labelled as A–H, with varying proportion of different phene states (b‐f) [Color figure can be viewed at wileyonlinelibrary.com]

**Figure 8:**
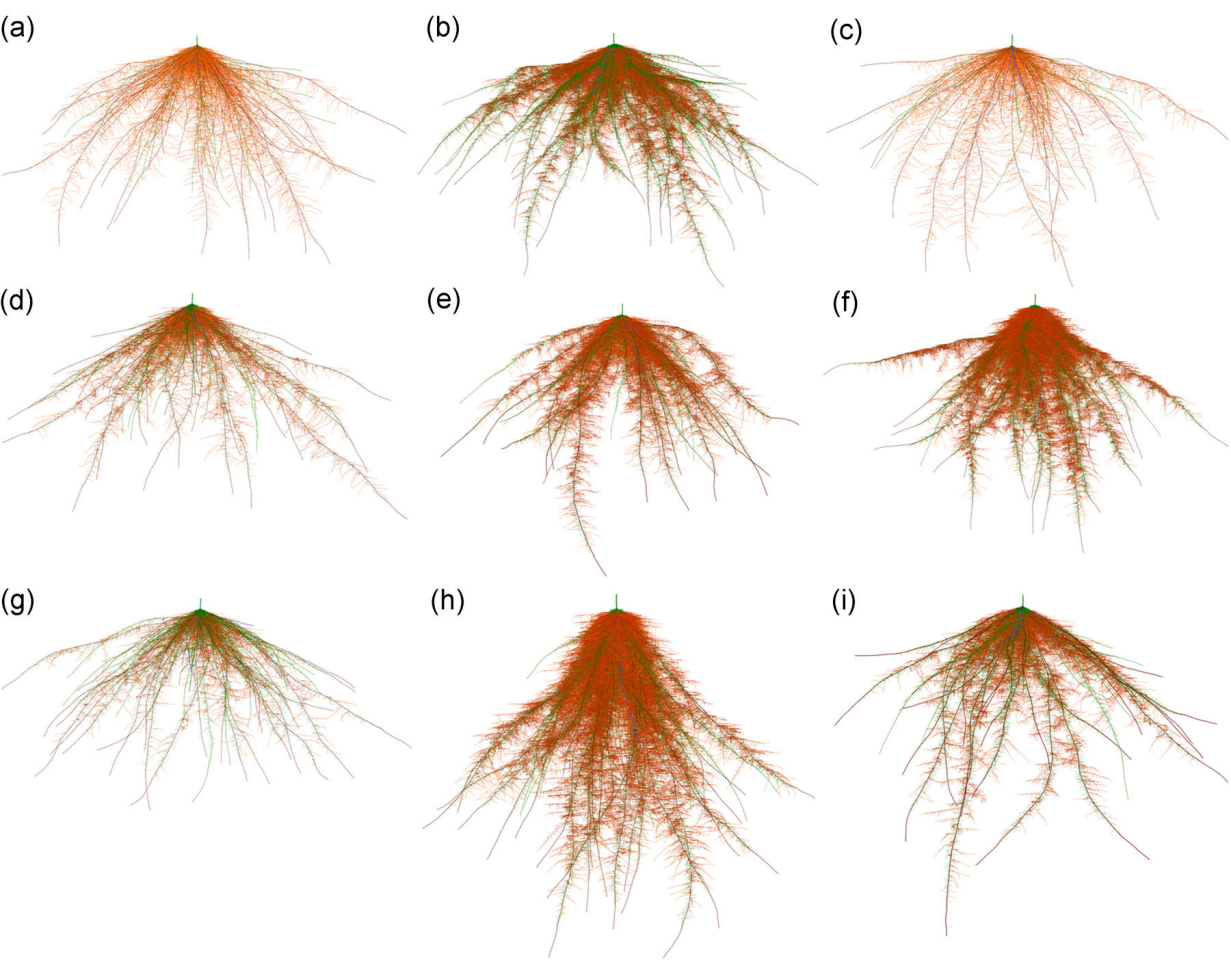
Visualization of representative root phenotypes corresponding to each of the eight identified Clusters (a–h) with the reference phenotype IR64 (i) at 30 DAG. Labels correspond with the cluster names [Color figure can be viewed at wileyonlinelibrary.com]

**Figure 9:**
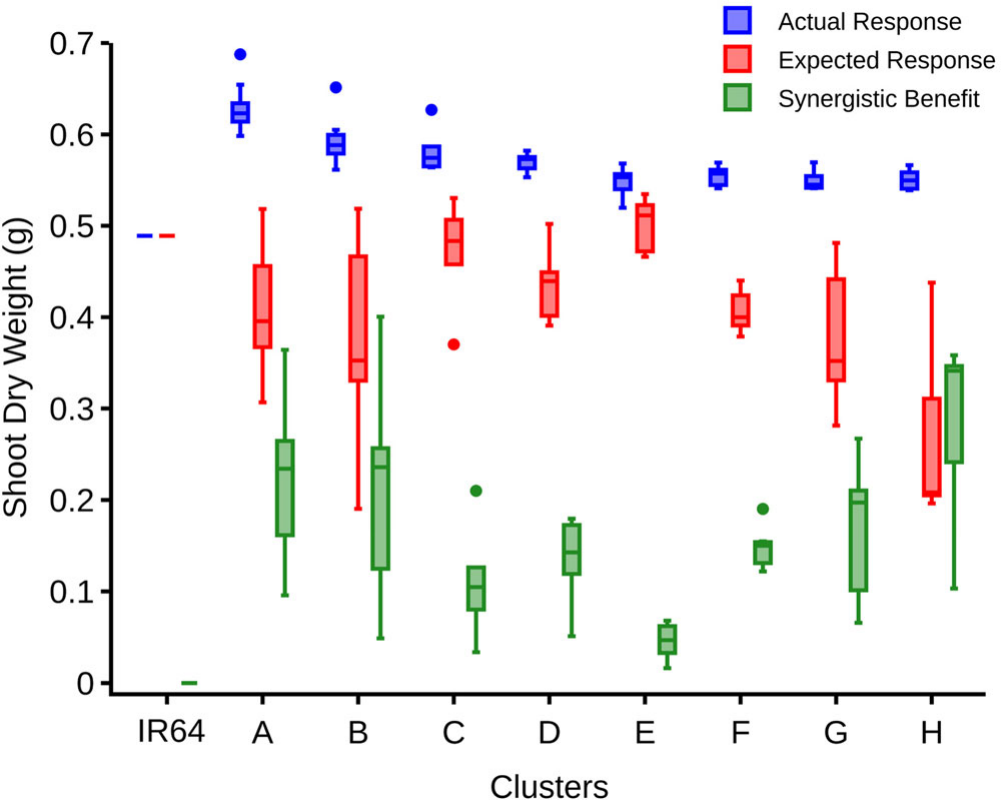
Synergistic benefits of phenotypes in each cluster. Actual and expected response (i.e., shoot biomass) of phenotypes in each cluster is highlighted along with their corresponding synergistic benefits [Color figure can be viewed at wileyonlinelibrary.com]

To pinpoint the optimal integrated phenotypes, the top 70 of the best performing phenotypes (i.e., with shoot biomass reduction ≤ 45% under low N supply) were selected for cluster analysis. These phenotypes were grouped into eight distinct clusters (A–H) in descending order of their corresponding shoot biomass gain in response to low N (Figures 7 and 8, Table 2). Two phene states predominated in at least six (i.e., A–D, G, H) of the eight clusters: shallow nodal root angle (i.e., 30°) and greater proportion (i.e., 100%) of small diameter nodal roots. Intermediate (45°) and steep (60°) nodal root angles and a moderately higher proportion (68%) of small diameter nodal roots segregated into three distinct clusters. The number of nodal roots, L‐type and S‐type lateral branching densities varied across the clusters while trading‐off with each other and balancing with the background phenotypes. With varying degree of benefits, all of the top 70 best performing phenotypes showed synergistic interactions among their underlying phenes (Figure 9). The varied synergistic benefits were distinct among the clusters with H and E being the most and the least synergistic clusters, respectively.

**Table 2:** Architectural features of the representative phenotypes from clusters along with reference IR64 (I)

Cluster	FLRBD (#/10 cm)	LRBD (#/10 cm)	NRA (°)	NRSD (%)	NRN (fold)	SDW (% reduction)
A	10	20	30	100	1.69	37.98
B	150	10	30	100	1.69	40.99
C	10	20	30	100	1	42.34
D	50	10	30	100	1.35	42.39
E	100	20	30	68	1	43.75
F	150	20	45	100	1.35	44.09
G	50	5	30	100	1.69	44.81
H	100	20	60	100	1.69	44.86
IR64	100	10	30	32	1	50.59

*Note*: The phene states are the mode from each cluster phenotype and the corresponding the reduction in shoot biomass is the median of the cluster.

**Figure 10:**
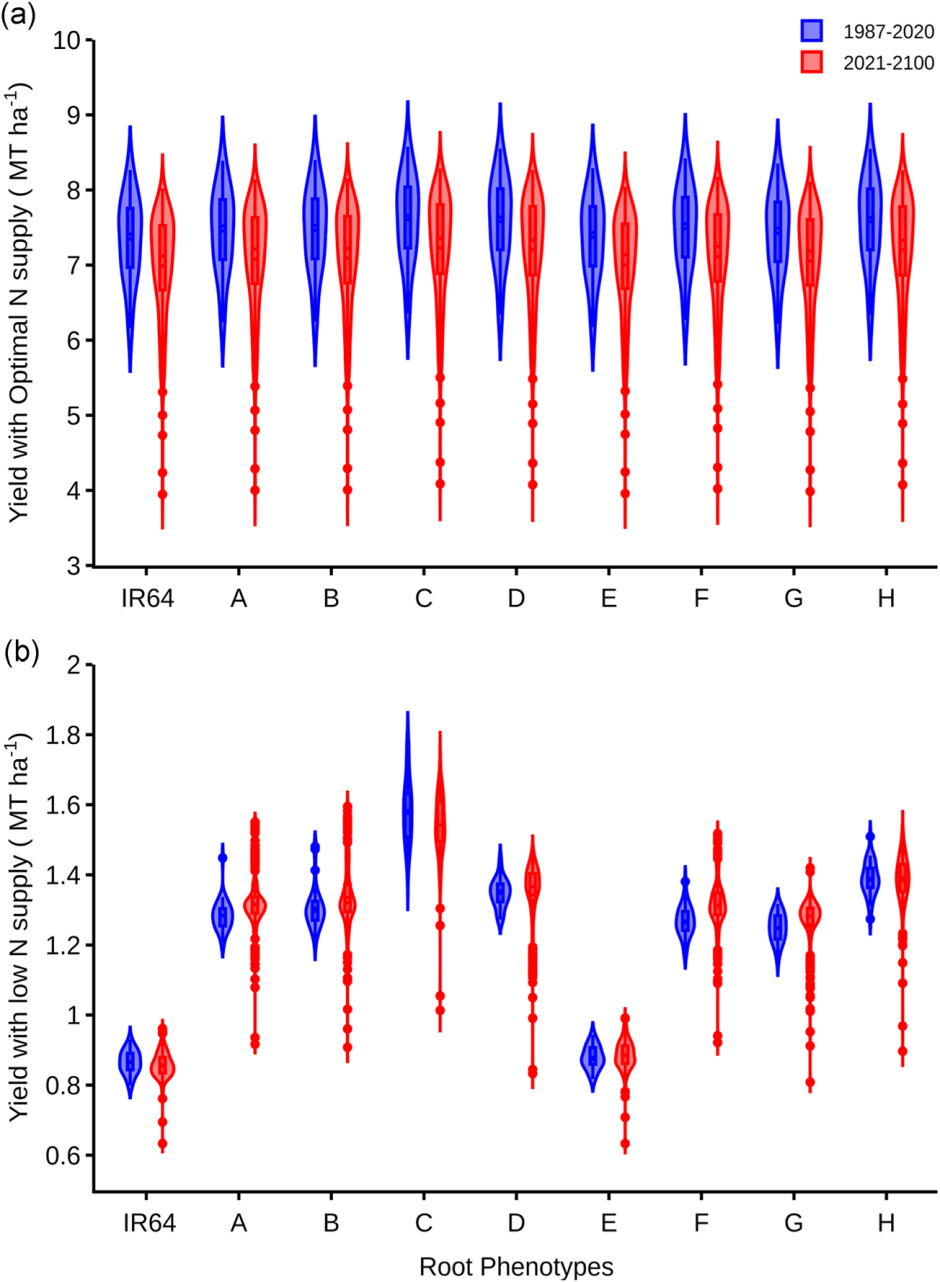
Predicted rice grain yield (with 14% moisture content) of representative root phenotypes corresponding to each of the eight identified Clusters (A–H) and the reference IR64 (I) over rainfed dry direct‐seeded conditions with (a) optimal (58–58–58 kg/ha) and (b) low (5.8–0–0 kg/ha) N fertilization with planting density of 20/m^2^. The phenotypes were simulated over 33 years of historic and 80 years of future dry season weather data from the IRRI research station, Los Baños, Philippines [Color figure can be viewed at wileyonlinelibrary.com]

Assessing the influence of varying root phenotypes on grain yield, eight phenotypes representative of each of the clusters along with the reference IR64 phenotype were simulated using the *ORYZA_V3* ecophysiology model with optimal and low N supply (Figure 10a,b). The yield of the reference cv. IR64 was predicted to be 7.15 Mt/ha with optimal N supply (Supporting Information [Link], [Link]). This is in agreement with actual 2016 dry season field observations at IRRI under optimal conditions. The predicted grain yield for the eight representative phenotypes (Clusters A–H), under optimal N were within the same range as IR64 (Figure 10a). In contrast, significant variability in predicted grain yield was observed among these phenotypes under low N (Figure 10b). Compared to IR64, these phenotypes were predicted to have between 2.2% and 83% greater grain yield under low N in both past and future climates. Among these, phenotype/Cluster C had 83% and 80% greater grain yield than IR64, over the past 30 years and the future 82 years of weather conditions, respectively, under low N fertilization. Cluster C consisted of six phenotypes with shallow small diameter nodal roots. The majority of these phenotypes have high lateral root densities, low fine lateral root densities, and nodal root numbers similar to the reference phenotype (Table 2).

## 4 DISCUSSION

Using an improved implementation of the functional–structural plant model *OpenSimRoot* for rice integrated with the crop agroecological model ORYZA, we evaluated the utility of root architectural phenes alone and in combination to predict optimal root architectural phenotypes for improved rice growth under varying N availability. For this evaluation, a ‘nonplastic’ root ideotype was adapted whereby the plant performance (i.e., plant biomass) would vary depending on the soil N availability but the defined state of a root phene in the phenotype remained indifferent to soil N availability. Eight sets of optimal root phenotypes were identified with improved growth under low N supply in rainfed dry‐directed seeded agroecologies. Most of these phenotypes had shallower root systems and a greater proportion of small diameter nodal roots. The number of nodal roots and lateral branching densities varied across these phenotypes while trading off with each other (i.e., when nodal root number is higher, then the lateral densities are lower or vice versa). The best phenotypes showed substantial phene synergism. Our results support the hypothesis that synergistic balancing of root architectural phenes is beneficial for plant growth under low N.

Root angle affects soil N capture in two ways: (1) by varying the synchrony between root foraging activity and N availability in space and time, and (2) by altering the dispersal of roots and thereby the extent of root competition within and among plants (Dathe et al., 2016; Lynch, 2019). Steeper root systems are less dispersed through the soil profile and are in very close proximity with each other, which leads to a counterproductive decline in N uptake efficiency and overall N capture even when N is more available at depth. The performance of a root phenotype thus depends on whether root foraging occurs in soil domains where N is available or will become available (Lynch, 2019). In rainfed DDSR, the availability of N is greater in topsoil domains because of slower leaching due to less rainfall over the dry season (Figure 5a,b). Therefore, shallower root systems coincided with N availability in the topsoil (Figure 5c,d) and had better performance under contrasting soil N regimes (Figure 3a).

Root diameter is an aggregate of several anatomical phenes including cortical cell size, cortical cell file number, and stele diameter. For simplicity, root diameter is treated as a phene in this study. Over evolution, thin roots have enabled plants to significantly improve their carbon cost of soil exploration (Ma et al., 2018). In general, rice has two classes of nodal roots with small and large diameters, occurring in varied proportions in different genotypes (Hoshikawa, 1989). When phenotypes with a greater proportion of small diameter nodal roots (from the main stem) were simulated, the reduced carbon cost improved shoot growth under low N. In contrast, phenotypes with a smaller proportion of small diameter nodal roots grew poorly (Figure 3b), presumably because the larger diameter increased nodal root sink strength. However, increased root diameter in rice has been associated with drought tolerance in other studies as it would enable penetration through hard soils and exploring deeper soil layers (Clark et al., 2008; Fukai & Cooper, 1995). We did not simulate soil hardness in this study, which would influence the fitness landscape of root diameter and elongation (Chimungu et al., 2015; Nguyen et al., 2018; Rich & Watt, 2013; Vanhees et al., 2020). Although root systems in which all nodal roots have small diameter were predicted to improve plant performance in response to low N availability, the utility of such a root system is yet to be explored in existing rice germplasm. The identification of phenotypes with varying proportions of small and large diameter nodal roots merits investigation.

Nodal roots successively emerge from nonelongated shoot nodes (Hoshikawa, 1989). The number of nodal roots emerging from each node acropetally increases, typically from 5 to >20, and varies among genotypes (Hoshikawa, 1989; Kawata et al., 1963; Matsuo & Hoshikawa, 1993). Our results suggest that increasing the number of nodal roots does not have any advantage in terms of shoot biomass gain under optimal and intermediate N supply. On the contrary, more nodal roots reduced shoot biomass gain under low N. Phenotypes with fewer nodal roots had better performance under low N supply (Figure 3c). This may be because increasing the number of roots increases competition for internal metabolic and external soil resources, thereby decreasing root growth, soil exploration, and capture of deep resources like N and water (Gao & Lynch, 2016; Postma et al., 2014; Saengwilai et al., 2014).

Lateral roots, both L‐type and S‐type, account for ∼90% of total root length but contribute <20% to the total root biomass, thereby increasing root surface area at low metabolic cost (Supporting Information 7; Nestler et al., 2016). Thus, phenotypes with greater lateral root density gained greater shoot biomass in low N soil (Figure 3d). However, altering the density of fine lateral roots did not significantly affect shoot biomass (Figure 3e). This can be ascribed to their extremely low cost of production and maintenance (Supporting Information 5; Wissuwa et al., 2020). Secondly, fine laterals have determinate growth, which along with intraroot competition limits their foraging capacity for mobile soil resources like nitrogen. Elevated intraroot competition due to greater lateral root density decreased the N uptake efficiency of the root system, particularly under greater soil N availabilities (Figure 4d,e). It is noteworthy that both lateral root classes together account for more than 50% of total nitrogen taken up by the root system, at low carbon cost (Supporting Information 5). A fine balance between the density of lateral roots and other architectural root phene states is thus essential for improved soil resource acquisition, including nitrogen.

The factorial simulation results demonstrate the importance of phene interactions in determining the fitness of an integrated root phenotype. Almost half (44%) of the 1024 root phenotypes evaluated showed significant synergism, meaning that their performance under low N exceeded the additive effects of their subtending phene states, using IR64 as the reference phenotype (Figure 6). A slightly larger portion, 52%, were simply additive, and a smaller portion, 4%, showed phene antagonism, whereby their performance was significantly worse than the expected effects of their subtending phene states (Figure 6). Notably, all phenotypic clusters with better performance under low N than IR64 were synergistic, including the three clusters with the best overall vegetative growth under low N (Clusters A and B in Figure 9). Indeed, considering only additive effects, only one cluster (Cluster E in Figure 9) exceeded the performance of IR64, and this by only a small amount. This may be related to the fact that IR64 is a widely adapted phenotype, which explains its history of being grown on millions of hectares across Asia (Mackill & Khush, 2018).

The importance of phene synergism for the performance of integrated root phenotypes under edaphic stress has been demonstrated before in silico. For example, the utility of root cortical aerenchyma for phosphorus (P) capture from low P soil in maize is 2.9 times greater in plants with increased lateral branching density (Postma & Lynch, 2011b). In *Arabidopsis*, the combination of increased root hair length, increased root hair density, increased proximity to the root tip at which root hairs begin to emerge, and increased number of trichoblasts, which are all coordinately regulated by low P availability, increased P capture by 371% more than their additive effects (Ma et al., 2001). In bean, multiple synergies are evident in the effect of axial root phenotypes on the acquisition of N and P (Rangarajan et al., 2018). Synergies have also been reported *in planta*: For example, shallow root growth angles and long root hairs are synergistic for P capture from low P soil in common bean (Miguel et al., 2015), and metaxylem anatomy and root depth are synergistic for drought adaptation in contrasting *Phaseolus* species (Strock et al., 2020). The importance of phene interactions for the performance of integrated root phenotypes has implications for breeding strategies to develop more stress‐tolerant cultivars. Ideotype breeding, informed by an understanding of how specific phene states interact in integrated phenotypes, may be more effective in this context than trait stacking, direct selection for yield, genomic selection, and other breeding strategies that do not consider the complexity of phene interactions.

K‐prototype clustering identified eight clusters (i.e., A–H) of optimal root phenotypes (Figure 7, Table 2). Of these, five clusters (A–D and G) have shallow nodal roots (30°) and a greater proportion (100%) of small diameter nodal roots. Clusters E, F and H are respectively distinct with a lower proportion (68%) of small diameter nodal roots, nodal root angle of 45° and 60° (Table 2). The number of nodal roots and lateral densities varied across each of these clusters. Rice root systems show large genotypic variation and extensive plasticity across germplasm adapted to different agroecosystems (O'Toole & Bland, 1987). For example, upland rice cultivars typically show deeper root systems and have larger axial root diameters compared to lowland rice cultivars (Kondo et al., 2000; O'Toole & Bland, 1987; Yoshida, Bhattacharjee et al., 1982; Yoshida & Hasegawa, 1982). Furthermore, plants may modify their root architectural phenes (e.g., through hormone regulation) in response to environmental conditions, such as nutrient status, to improve their fitness.

Coupling *OpenSimRoot/Rice* with the ecophysiological rice crop model *ORYZA_v3* is one of the novel features of this study, enabling the heuristic estimation of yield performance of the representative cluster phenotypes under low N supply in rainfed DDSR agroecologies over past and future weather conditions. Predicted grain yield of the optimal phenotypes were significantly greater than the reference phenotype in low N except for one phenotype (E). This may be a result of the relatively high carbon allocation to roots over 30 DAG in phenotype E (Supporting Information 6). Yield losses in low‐input rice production systems are inevitable due to various environ–socioeconomic factors. The predicted phenotype aims at reducing the yield loss. Notably, the predicted optimal phenotypes had relatively less carbon allocation to roots compared to the reference phenotype, because of their reduced metabolic demand of root production and maintenance. Besides yield estimations, this model coupling effort underlined the importance of shoot and root carbon partitioning for plant adaptations to edaphic stress, particularly in early growth stages (Lynch, 2007).

Root carbon investment, nitrogen acquisition and shoot biomass gain are interdependent response variables, contributing to plant adaptation to low N availability. Root carbon investment accounts for construction, maintenance, and resource uptake cost of the root system. Root carbon cost largely depends on the phenes underlying the corresponding phenotype. Depending on the phene states, different phenotypes acquire different amounts of soil nitrogen, thereby either delaying, avoiding, or mitigating low N stress and in turn, accumulating maximum possible shoot biomass. Despite similar root carbon cost, phenotypes may acquire different amounts of nitrogen due to their varying soil foraging capacities and in turn, accumulate variable shoot biomass in response to low N (Supporting Information 7).

The reference *OSR/Rice* simulation for 30 DAG led to a root system consisting of 0.1 million root segments, which is ∼3, 40.5 and 4.2 times greater than the simulated 30‐day old root systems for maize, barley, and common bean, respectively. Consequently, simulating the rice root system with the current version of the *OpenSimRoot* platform is relatively computationally challenging, which constrained both the length of the simulations (i.e., beyond 30 DAG) and the size of our factorial simulations. However, compared to previous *SimRoot* studies (Dathe et al., 2016; Rangarajan et al., 2018), the fitness landscape for more than three architectural root phenes was explored for the first time in this study. Notably, this study offers an in silico platform to access *n*‐number of potential rice root phene combinations, including plastic expression of different root phenes combinations, under different environmental scenarios that may not necessarily exist now but could be developed through breeding. It is anticipated that this study will inform empirical studies that ultimately could promote the development of rice genotypes more tolerant to edaphic stresses, including low N supply.

This study attempts to nondynamically link a plant‐scale model (i.e., *OpenSimRoot)* and field‐scale crop growth model (i.e., *ORYZA_v3)*. Though both these models have common terminologies for some variables such as crop biomass, leaf area index, water uptake and nitrogen uptake, there are inconsistencies in the magnitude of the variables accounting for crop nutrient stress, namely water and nutrient uptake. This is particularly due to the difference in spatial scales at which the two models operate. Given such challenges, the development of computational tools for the integration of independently developed models at different spatiotemporal scales, such as *OpenSimRoot* and *ORYZA_V3*, would facilitate in silico phenotypic evaluation of large breeding populations. This would certainly require benchmarking of the models and the ontologies of the model variables and parameters. Recently, model integration initiatives have been started (Benes et al., 2020; Marshall‐Colon et al., 2017) via the development of polyglottic multiscale model integration framework, namely yggdrasil (Lang, 2019) and benchmarking efforts have been initiated by the crop modelling (Confalonieri et al., 2016) and functional–structural root architecture modelling (Schnepf et al., 2020) communities.

## 5 CONCLUSIONS

Optimizing the allocation of internal resources to optimize the acquisition of external resources presents opportunities for crop improvement. Exploring this paradigm, an upgraded functional–structural model *OpenSimRoot/Rice* was employed to identify optimal root phenotypes informing the design of root systems for stress‐adapted rice, particularly for low N supply. Results support the hypothesis that synergistic balancing of root phenes is beneficial for plant growth with low N supply. Shallower nodal root angle favoured colocalizing root foraging with N availability in dry seasons. The greater proportion of nodal roots with smaller diameter and fewer number of nodal roots reduced metabolic costs. Higher density of L‐type lateral roots enhanced N foraging. Synergistic co‐optimization of these phenes improved plant performance under low N. These findings led to physically contrasting but functionally similar phenotypes. From the 1024 integrated phenotypes we assessed, eight groups were identified with biomass accumulation greater than the reference phenotype under low N. The benefits gained by these superior phenotypes can be attributed to the synergistic interactions among their underlying phenes. Representative phenotypes of these groups had up to 83% greater predicted grain yield compared to the reference IR64 with low N supply under rainfed dry direct‐seeded conditions over historic climates, and 80% over projected future climates. These phenotypes merit consideration as root ideotypes for breeding low N‐adapted rice cultivars.

## CONFLICT OF INTERESTS

The authors declare that there are no conflict of interests.

## Supporting information

Supporting information.

Supporting information.

## Data Availability

Data will be available from the corresponding author on request.
