# Supplementary material for: Integrated root phenotypes for improved rice performance under low nitrogen availability
Source: Plant Cell Environ. 2022 Feb 23;45(3):805–22. doi: 10.1111/pce.14284 (PMC9303783; doi:10.1111/pce.14284)

**Supplementary Information 1**

1. **Link to the executable code of OpenSimRoot version used in this study and the input run file for OpenSimRoot/Rice**

<https://doi.org/10.5281/zenodo.4662125>

*Table S1.1. References for parameters used OpenSimRoot/Rice*

| Parameter sets | References |
| --- | --- |
| Root morphology  (root class, angle, diameter,  number, lateral density) | - Iljima et al., 1998 - Moldenhauer KAK and Gibbons JH. 2003 - Kato et al. 2006 - Brar DS 2007 - Périn et al, 2007 - Shashidhar et al., 2012 - Grondin et al, 2016 - Vejchasarn et al.,2016 - Gu et al 2017 |
| Leaf area development | - Luquet et al., 2006 - Hairmansis et al., 2014 - Vikram et al., 2015 |
| Optimal and minimal  nitrogen concentrations | - Mosse J. 1990 - Dobermann and Fairhurst, 2000 - <https://www.haifa-group.com/rice-0/crop-guide-rice-fertilizer-parameters#{E444C0EF-DBB0-4F61-A1ED-A9B28C53D2B2}> - <https://sites.google.com/a/irri.org/oryza2000/about-oryza-version-3> |
| Root hair density and growth | - Vejchasarn et al., 2016 |
| Root respiration | - Supplementary information 1B |
| Carbon module | - Choudhury B., 2001 - Luquet et al., 2006 - <https://sites.google.com/a/irri.org/oryza2000/about-oryza-version-3> |
| Aerenchyma formation | - Henry et al., 2012 - Grondin et al, 2016 - Kadam et al., 2015 |
| Radial and Axial  Hydraulic Conductivity | - Miyamoto et al. 2001 - Ranathunge et al. 2003 - Schreiber et al., 2005 - Kadam et al. 2015 |
| Tillering | - Jaffuel S and Dauzat J, 2005 - Luquet et al., 2006 |
| Soil hydrology | - <https://sites.google.com/a/irri.org/oryza2000/about-oryza-version-3> |
| Weather data | - IRRI, Los Baños, weather station (2016) |
| Uptake kinetics | - Youngdahl LJ et al.,1982 - Hasegawa and Ichii 1994 - Sands & Smethurst 1995 - Kronzucker HJ et al 2000 - <https://www.pc-progress.com/Downloads/Miscellaneous/Nitrate_uptake_kinetics_extended_list.xls> |

**Footnotes** :

**^§^** Other remaining parameters were adapted from pervious OpenSimRoot implementation (Schneider et al., 2017).

**^§^** Parameters converted to appropriate units and at times were rationally adjusted based on empirical understanding of rice roots.

1. **Rice root respiration measurements**

Rice (Oryza sativa cv. *IR64*) was used in the experiments. Dehusked rice seeds were soak overnight in tap water and pre-germinated on petri dish for 3 days. 30 uniformly grown seedlings were then transferred to the 100-litre tank situated in a greenhouse at Pennsylvania State University in University Park (40.801955°N, 77.862544°W). Plants were grown between August and September 2017 with under a 12-h photoperiod, average day/night temperatures of 30/24°C and 55% relative humidity. Natural light was supplemented 110 μmol photons m^−2^ s^−1^ from LED Illumitex ES2 lights (Illumitex). The nutrient solution consisted of a half-strength solution as described by Yoshida et al. (1976), containing 0.7125 mM NH_4_NO_3_, 0.2565 mM K_2_SO_4_, 0.499 mM CaCl_2_, 0.8215 mM MgSO_4_, 0.0375 µM (NH_4_)_6_Mo_7_O_24_, 0.0045 mM MnCl_2_, 0.01 µM H_3_BO_3_, 0.07 µM CuSO_4_, 0.07 µM ZnSO_4_, 0.0625 mM EDTA-Fe, and 0.1615 mM NaH_2_PO_4_. The pH of the solution was adjusted to 5.5-5.6. The solution was continuously aerated and renewed every 3 days. At 30 DAG, respiration rates for root segments corresponding to different root classes and age were determined. For each plant, thin and thick nodal roots were identified by eyeballing. 4-5 root segments each of 4 cm in length from the tip and from the base of nodal roots were sequentially collected. These root segments were briefly surface dried using paper towel, lateral roots were manually removed and then placed in the respiration chamber. From each plant, a bunch of lateral roots (including L-types and S-types) were randomly taken for the measurements. Respiration rates were measured using a Li-Cor 6400 gas-exchange system with a modified respiration chamber (Li-Cor). Measurements were performed under ambient greenhouse conditions, with the sealed chamber being submerged in another tank containing nutrient solution. This was done to maintain the temperature of the chamber close to that experienced by the roots in the nutrient solution. The baseline sample chamber and reference chamber CO2 concentration were set to 400 μmol mol−1. Following respiration measurements, each sample was dried using a Leica EM CPD300 critical point dryer (Leica Microsystems) and then weighed in order to estimate specific root respiration rates (Figure S1.1, below)


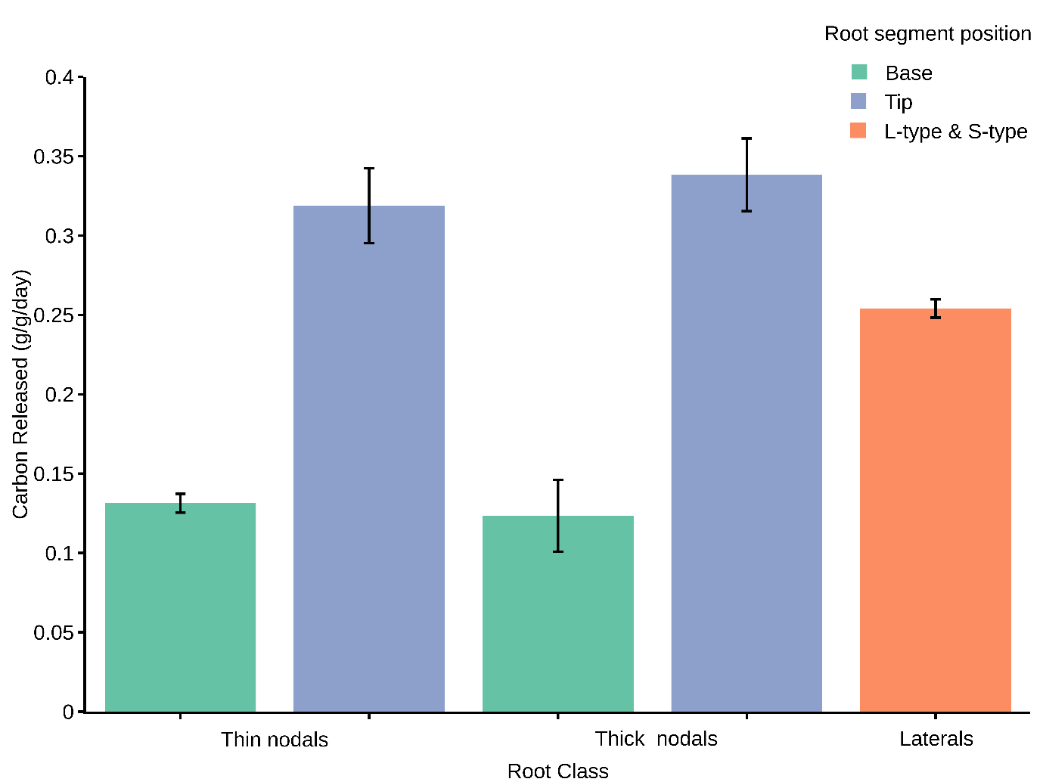


*Figure S1.1. Specific root respiration rate for Rice cv. IR64. Data shown are means ±*

*SE for 30 plants.*

1. **Reference for supplementary Information 1 :**

**Brar, DS** (2007) Rice genetics V. Manila, IRRI; Singapore: World Scientific.

**Choudhury BJ** (2001) Modeling radiation- and carbon-use efficiencies of maize, sorghum, and rice. Agricultural and Forest Meteorology.106(4):317-330

**Dobermann A**, Fairhurst T (2000) Rice : Nutrient Disorders & Nutrient Management. Potash & Phosphate Institute and International Rice Research Institute

**Grondin A**, Mauleon R, Vadez V, Henry A (2016). Root aquaporins contribute to whole plant water fluxes under drought stress in rice (Oryza sativa L.). Plant Cell Environ. 39(2):347-65.

**Gu, D**., Zhen, F., Hannaway, D. B., Zhu, Y., Liu, L., Cao, W., & Tang, L. (2017). Quantitative Classification of Rice (Oryza sativa L.) Root Length and Diameter Using Image Analysis. PloS one, 12(1), e0169968.

**Hairmansis A**, Berger B, Tester M, Roy SJ. (2014) Image-based phenotyping for non-destructive screening of different salinity tolerance traits in rice. Rice (N Y) 7(1):16.

**Hasegawa H** and Ichii M, (1994) Variation in Michaelis-Menten Kinetic Parameters for Nitrate Uptake by the Young Seedlings in Rice (Oryza sativa L.), Japanese Journal of Breeding, 44(4):383-386

**Henry A,** Cal AJ, Batoto TC ,Torres RO ,Serraj R . 2012. Root attributes affecting water uptake of rice (Oryza sativa) under drought. Journal of Experimental Botany63, 4751–4763.

**Henry A,** Swamy BP, Dixit S, Torres RD, Batoto TC, Manalili M, Anantha MS, Mandal NP, Kumar A (2015) Physiological mechanisms contributing to the QTL-combination effects on improved performance of IR64 rice NILs under drought. J Exp Bot. 66(7):1787-99.

**Iljma M**, Oribe Y, Horribe Y, Kono Y (1998) Time Lapse Analysis of Root Elongation Rates of Rice and Sorghum During the Day and Night. Annals of Botany 81(5):603–607

**Jaffuel, S**., & Dauzat, J. (2005). Synchronism of leaf and tiller emergence relative to position and to main stem development stage in a rice cultivar. Annals of botany, 95(3), 401–412.

**Kadam NN**, Yin X, Bindraban PS, Struik PC, Jagadish KS (2015) Does morphological and anatomical plasticity during the vegetative stage make wheat more tolerant of water deficit stress than rice? Plant Physiol 167: 1389–1401

**Kato Y,** Abe J, Kamoshita A, Yamagishi J (2006). Genotypic Variation in Root Growth Angle in Rice (Oryza sativa L.) and its Association with Deep Root Development in Upland Fields with Different Water Regimes. Plant Soil 287, 117–129

**Kondo M**, Aguilar A, Abe J, Morita S (2000) Anatomy of Nodal Roots in Tropical Upland and Lowland Rice Varieties. Plant Production Science 3: 437-445

**Kronzucker HJ**, Glass ADM, Siddiqi MY, Kirk GJD (2000). Comparative kinetic analysis of ammonium and nitrate acquisition by tropical lowland rice: implications for rice cultivation and yield potential. New Phytologist 145: 471–476.

**Xu L**, Henke M, Zhu J, Kurth W, Buck-Sorlin G (2011) A functional–structural model of rice linking quantitative genetic information with morphological development and physiological processes, Annals of Botany, 107(5): 817–828

**Luquet D**, Dingkuhn M, Kim H, Tambour L, Clement-Vidal A (2006). EcoMeristem, a model of morphogenesis and competition among sinks in rice. 1. Concept, validation and sensitivity analysis. Funct Plant Biol. 33(4):309-323.

**Miyamoto N**, Steudle E, Hirasawa T, Lafitte R (2001). Hydraulic conductivity of rice roots. J Exp Bot. 52(362):1835-46.

**Moldenhauer KAK** and Gibbons JH. (2003) Rice Morphology and Development. Chapter 2.1. Rice: Origin, History, Technology, and Production. Eds. Wayne S C and Dilday R H. John Wiley & Sons.

**Mosse J** (1990) Nitrogen-to-protein conversion factor for ten cereals and six legumes or oilseeds. A reappraisal of its definition and determination. Variation according to species and to seed protein content. Journal of Agricultural and Food Chemistry 38: 18-24

**Périn C**, Rebouillat J, Brasileiro AMC, Diévart A, Gantet P, Breitler JC, Johnson AAT, Courtois B, Ahmadi N, Raissac Md, Luquet D, Conte M, This D, Pati PK, Le QH, Meynard D, Verdeil JL, Guiderdoni E (2007) Novel insights into the genomics of rice root adaptive development. *In* Rice Genetics V, pp 117-141

**Ranathunge K**, Steudle E, Lafitte R (2003). Control of water uptake by rice ( Oryza sativa L.): role of the outer part of the root. Planta. 217(2):193-205.

**Sands PJ,**  Smethurst PJ (1995) Modelling Nitrogen Uptake in Ingestad Units Using Michaelis-Menten Kinetics. Functional Plant Biology 22, 823-831.

**Schneider HM**, Postma JA, Wojciechowski T, Kuppe C, Lynch JP (2017) Root Cortical Senescence Improves Growth under Suboptimal Availability of N, P, and K. Plant Physiol 174: 2333-2347

**Schreiber L**, Franke R, Hartmann KD, Ranathunge K, Steudle E (2005) The chemical composition of suberin in apoplastic barriers affects radial hydraulic conductivity differently in the roots of rice (Oryza sativa L. cv. IR64) and corn (Zea mays L. cv. Helix), Journal of Experimental Botany 56(415):1427–1436

**Shashidhar HE**, Henry A, Hardy B, editors. (2012). Methodologies for root drought studies in rice. Los Baños (Philippines): International Rice Research Institute pp 65

**Vejchasarn P**, Lynch JP, Brown KM (2016). Genetic Variability in Phosphorus Responses of Rice Root Phenotypes. Rice (N Y). 9(1):29

**Vikram P**, Swamy BP, Dixit S, Singh R, Singh BP, Miro B, Kohli A, Henry A, Singh NK, Kumar A (2015). Drought susceptibility of modern rice varieties: an effect of linkage of drought tolerance with undesirable traits. Sci Rep 5:14799

**Yoshida, S.,** Forno, D., Cock, J., and Gomez, K. (1976). Laboratory Manual for Physiological Studies of Rice, 3rd ed. (Manila, The Philippines: The International Rice Research Institute).

**Youngdahl LJ**, Pacheco R, Street JJ, Vlek PLG (1982). The kinetics of ammonium and nitrate: uptake by young rice plants. Plant Soil. 69:225–232.

**Supplementary information 2:**

Interaction among different phene combinations for shoot biomass gain in response to soil low N availability. Here is link to the R-script for calculating expected response of a phene combination along with the corresponding input and output files:

<https://doi.org/10.5281/zenodo.4662116>

**Supplementary information 3:**

*OpenSimRoot/Rice-ORYZA model coupling approach and evaluation for rice growth and yield simulation*

The effect of root phenotypes on rice grain yield was estimated by coupling the *OpenSimRoot*/*Rice* (*OSR/Rice*) model with the *ORYZA_v3* model.

Figure 1: Steps involved in non-dynamic coupling of *OpenSimRoot/Rice* and *ORYZA* model. OSR, OpenSimRoot/Rice

***Step 1 Parameterizing ORYZA model with the output from OpenSimroot/Rice model for each of the eight cluster phenotypes under optimal and low N supply.***

*OpenSimRoot*/Rice outputs including leaf area index, leaf area, specific carbon allocation to shoot, leaf, stem and roots, root biomass at different depths, and minimum and maximum nitrogen content for a single rice plant over 30 days after germination (DAG) were converted to input parameters for ORYZA model capturing 200,000 plants per hectare. These parameters were root front velocity (GZRT, daily rate of root depth progression throughout soil profile in m d^-1^), fraction of plant biomass allocated to shoot (FSHTB), fraction of shoot biomass allocated to leaves and stem (FLVTB and FSTTB), and daily LAI. It is assumed that by 30 DAG the rice root system has reached its optimum development beyond which no significant root growth occurs. The objective is then to inform the ORYZA model on plant trait variation among the representative phenotypes and determine the impact of different root phenotypes on the final grain yield under low and optimal nitrogen supply or fertilization.

***Step 2 Calibration of ORYZA parameters to fit the biomass for each cluster phenotypes simulated by OpenSimRoot over 30 DAG under optimal and low N supply.***

The ORYZA model crop parameters for FSHTB, FLVTB, FSTTB, GZRT and soil parameters of nitrogen supply were calibrated to minimize the root mean square error between simulations outputs from the two models for total above-ground and root biomass. Statistical comparison of these variables along with water and nitrogen uptake were used to evaluate ability of the ORZYA model to accurately capture the impact of varying root phenotypes on crop growth, despite non-explicit representation of roots. Steps 1 and 2 were carried out to ensure and provide confidence in the ability of ORYZA to simulate rice growth with different root phenotypes by assimilating the early growth stage inputs from *OpenSimRoot*.

***Step 3 Evaluation of the ORYZA model in simulating grain yield for each phenotype cluster under optimal and low nitrogen supply.***

Using the crop parameters for reference cv. IR64 from *OpenSimRoot/Rice*, the ability of the ORYZA model to simulate grain yield under optimal and low N supply was evaluated. Statistical parameters comparing the observed and the simulated yield were estimated, thereby confirming the accuracy underlying the coupling of the two models in predicting final grain yield for the identified root phenotypes. Grain yield data collected from field experiments conducted in 2016 wet season and 2016-2017 dry season under high and low nitrogen application at IRRI, Philippines, were used.

***Step 4 Scenario simulation with the ORYZA model for each phenotype cluster***

The ORYZA model was simulated for historic and near future weather conditions to simulate rice yield for the eight different root phenotypes identified in this work. The past 33 years (i.e., from 1987 to 2020) were considered for the historic period. Years from 2020 to 2100 were considered for the near future period. Predicted yield for each phenotype was compared with predicted yield of the reference variety cv. IR64 over both past and future weather conditions.

***ORYZA prediction for 30-day root growth, water uptake, N uptake using calibrated parameters estimated for each phenotype cluster from OpenSimRoot simulations.***

Under low and high N, agreement between ORYZA and *OpenSimRoot/Rice* outputs was acceptable with RMSE ranging from 7.0 to 23.0%. This indicates goodness of fit between the outputs from the two models for shoot and root biomass, and water and nutrition uptake during the first 30 days of crop growth. Deviation between the root biomass simulated by the two models under high N were largest among the objective variables, with an overestimation of more than 50% of root biomass simulated by the model ORYZA compared with the root biomass simulated by *OpenSimRoot/Rice*. This deviation was observed mainly for root biomass in the first soil layer as illustrated for the variety of reference IR64 in the ***Figure S1.*** (below). However, a good agreement between the two models was observed under low N conditions (*Table S1 and Figure S1*). Notably, root biomass estimated from *OpenSimRoot/Rice* is for individual plants over 25 cm x 20 cm spacing whereas in ORYZA neighbouring plants would contribute to the total root biomass exploring the same space. This difference in spatial representation may contribute to the large error between the two models in simulating total root biomass.


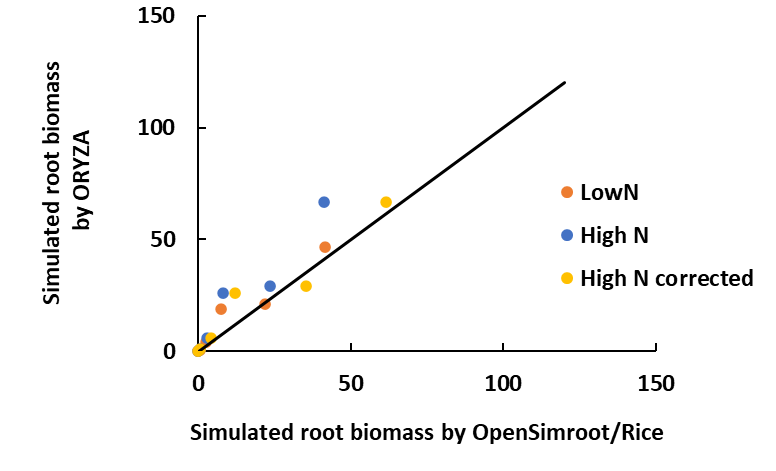
Using the crop parameters estimated from *OpenSimroot/Rice* for the variety cv. IR64, the ORYZA model presented acceptable ability in simulating rice yield under high and low N supply ***Figure S2*** (below). The accuracy of the ORYZA model to simulate grain yield using inputs from *OpenSimRoot/Rice* confirmed that the present approach in coupling the two models presented acceptable ability to translate the effect of variation of root traits among genotype to variation in grain yield.

***Figure S1*** IR64 root biomass simulated by ORYZA and *OpenSimRoot/Rice* under high and low N for a crop sown on January 22, 2016. ‘High N corrected’ values are from root biomass simulated by ORYZA and simulation outputs by OpenSimRoot accounting for neighbouring plant contributing to total root biomass within the soil volume explored by individual plants at a 25 x 20 cm spacing.


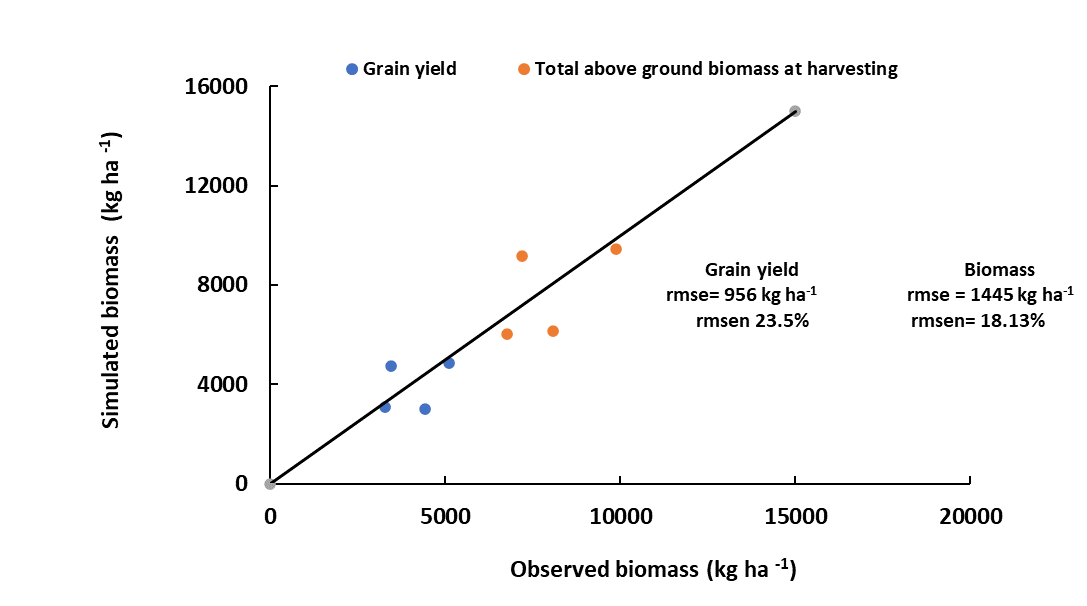
.

***Figure S2*** Observed vs simulated grain and total above ground biomass for IR64 under optimal and low N supply. Simulated yield and biomass were generated by the ORYZA model using inputs from *OpenSimRoot/Rice*. Observed IR64 yield and biomass is from an experimental field at IRRI with optimal and low N application during the wet season 2016 and dry season 2016-2017.

***Table. S1*** Comparison of crop biomass, root biomass, water uptake, N uptake from *OpenSimRoot/Rice* and ORYZA models for the 8 cluster phenotypes under a) optimal, and b) low soil N supply

a.

| Parameters | *OpenSimRoot/Rice* | *OpenSimRoot/Rice*-ORYZA | RMSE_n (%) |
| --- | --- | --- | --- |
| WAGT | 177.80±0.13 | 211.34± 18.9 | 21.35 |
| WRT | 83.11±19.7* | 96.13± 18.5 | 17.22* |
| Water uptake | 94.8±0 | 95.78±0.23 | 7.4 |
| N uptake | 27.48± 1.88 | 17.67±0.28 | 10.4 |

b.

| Parameters | *OpenSimRoot/Rice* | *OpenSimRoot/Rice*-ORYZA | RMSE_n (%) |
| --- | --- | --- | --- |
| WAGT | 108.77±9.53 | 116.0± 18.5 | 13.96 |
| WRT | 82.98±20.25* | 71.59± 11.80 | 18.82* |
| Water uptake | 77.45±4.85 | 94.6±0.4 | 23.0 |
| N uptake | 3.27±0.20 | 2.79±0.44 | 18.82 |

*RMSE_n with correction of the *OpenSimRoot/Rice* root biomass simulations by 1.5 to render value of root biomass at field scale from the single plant scale simulation.

**Supplementary information 4:**

Link to the OpenSimRoot/Rice - IR64 simulation of root growth over 30 DAG

<https://doi.org/10.5281/zenodo.4560508>

**Supplementary Figure 5**

Influence of varying lateral and fine lateral root branching densities on their contribution to root dry weight, total root length and nitrogen uptake in 30-day old rice plant under low N supply.


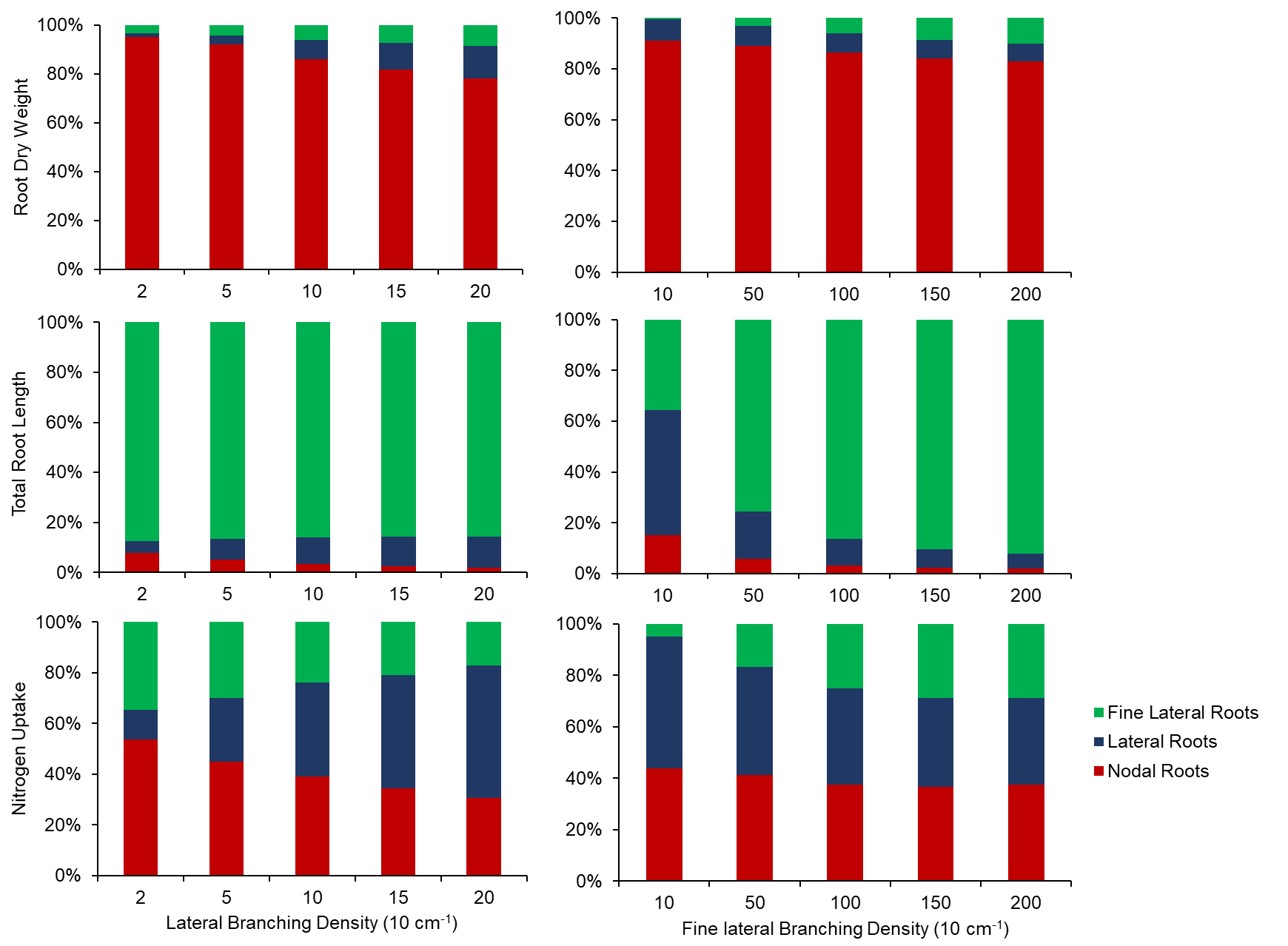


**Supplementary Figure 6**

Percentage of carbon allocation to the shoot from the total carbon available for growth in each cluster (A-H) and reference IR64 (I) root phenotype under low N supply.


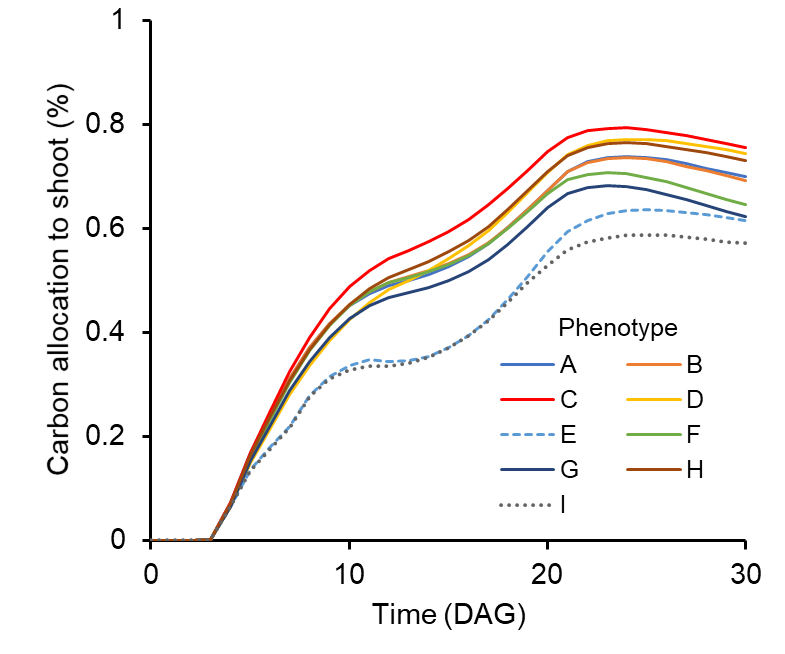


**Supplementary Figure 7**

Carbon invested in roots, shoot biomass gain and nitrogen acquired over 30 DAG by the simulated 1024 phenotypes in response to low N supply.


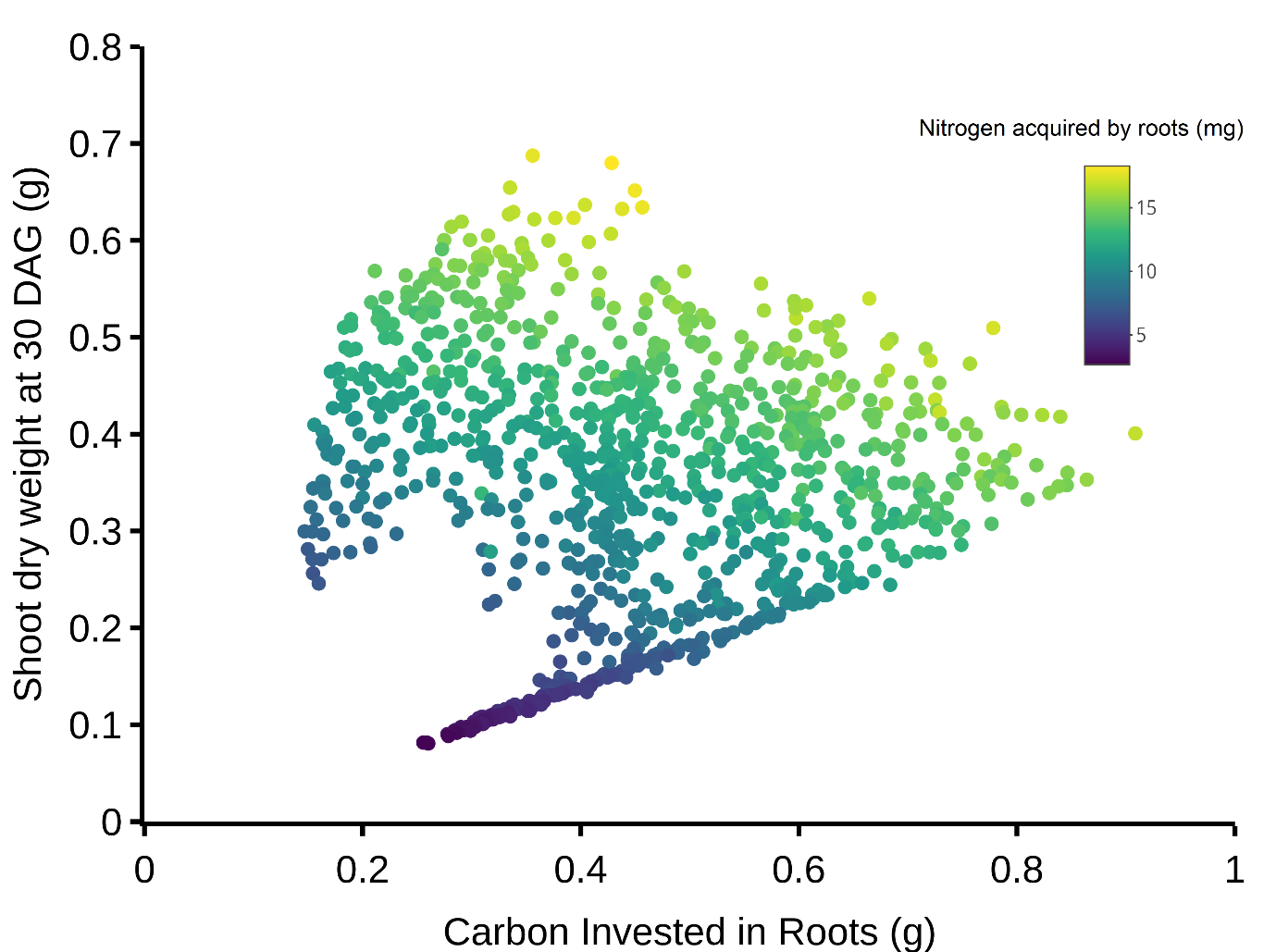

Supplement: Supplementary file 1 — Supporting information. [file PCE-45-805-s001.docx]
