# Supplementary material for: Integrated root phenotypes for improved rice performance under low nitrogen availability
Source: Plant Cell Environ. 2022 Feb 23;45(3):805–22. doi: 10.1111/pce.14284 (PMC9303783; doi:10.1111/pce.14284)
Supplement: Supplementary file 2 — Supporting information. [file PCE-45-805-s002.docx]

**Supplemental Data**

The following materials are available in the online version of this article.

Supplementary Information 1: Executable code of the OpenSimRoot version of employed in this study, input file and parameters references.

*Supplementary Information 2:* Interaction among different phene combinations for shoot biomass reduction in response to soil low N availability.

*Supplementary Information 3*: OpenSimRoot/Rice-ORYZA model coupling approach and evaluation for rice growth and yield simulation

*Supplementary Information 4:* OpenSimRoot/Rice simulation video of root growth over 30 DAG.

*Supplementary Information 5*: Contribution of lateral and fine lateral roots in root dry weight, root length and nitrogen uptake under low N supply.

*Supplementary Information 6:* Shoot carbon allocation in each representative cluster phenotypes over 30 DAG under low N condition.

*Supplementary Information 7:* Root carbon investment, shoot biomass gain and nitrogen acquired by the simulated 1024 phenotypes in response to low N supply.
